# Bacteria differently deploy type-IV pili on surfaces to adapt to nutrient availability

**DOI:** 10.1038/npjbiofilms.2015.29

**Published:** 2016-02-24

**Authors:** Lei Ni, Shuai Yang, Rongrong Zhang, Zhenyu Jin, Hao Chen, Jacinta C Conrad, Fan Jin

**Affiliations:** 1 Hefei National Laboratory for Physical Sciences at the Microscale, Department of Chemical Physics, Department of Polymer Science and Engineering, CAS Key Laboratory of Soft Matter Chemistry, University of Science and Technology of China, Hefei, China; 2 Coordination Chemistry Institute and the State Key Laboratory of Coordination Chemistry, School of Chemistry and Chemical Engineering, Nanjing University, Nanjing, China; 3 Chemical and Biomolecular Engineering Department, University of Houston, Houston, TX, USA

## Abstract

The structure of bacterial biofilms depends on environmental conditions, such as availability of nutrients, during biofilm formation. In turn, variations in biofilm structure in part reflect differences in bacterial motility during early biofilm formation. *Pseudomonas aeruginosa* deprived of nutrients remain dispersed on a surface, whereas cells supplemented with additional nutrients cluster and form microcolonies. At the single-cell scale, how bacteria modify their motility to favour distinct life cycle outcomes remains poorly understood. High-throughput algorithms were used to track thousands of *P. aeruginosa* moving using type-IV pili (TFP) on surfaces in varying nutrient conditions and hence identify four distinct motility types. A minimal stochastic model was used to reproduce the TFP-driven motility types. We report that *P. aeruginosa* cells differently deploy TFP to alter the distribution of motility types under different nutrient conditions. Bacteria preferentially crawl unidirectionally under nutrient-limited conditions, but preferentially stall under nutrient-supplemented conditions. Motility types correlate with subcellular localisation of FimX, a protein required for TFP assembly and implicated in environmental response. The subcellular distribution of FimX is asymmetric for unidirectional crawling, consistent with TFP assembled primarily at the leading pole, whereas for non-translational types FimX expression is symmetric or non-existent. These results are consistent with a minimal stochastic model that reproduces the motility types from the subcellular average concentration and asymmetry of FimX. These findings reveal that *P. aeruginosa* deploy TFP symmetrically or asymmetrically to modulate motility behaviours in different nutrient conditions and thereby form biofilms only where nutrients are sufficient, which greatly enhances their competitive capacity in diverse environments.

## Introduction

Biofilms, surface-associated communities of bacteria surrounded by a protective matrix composed of extracellular polymers, are ubiquitously found in environmental, technological and medical settings.^1–3^ Bacteria living in these complex differentiated multicellular communities can exhibit increased virulence and resistance to stress. The biofilm state may thus provide advantages to the enclosed bacteria that are not available to individual planktonic cells, especially in harsh or unfavourable environments.^4^ Bacteria start to form biofilms in response to a variety of environmental cues, including nutrient availability, solute concentration, temperature and hydrodynamic forces.^5–12^ Natural biofilms are often found in heterogeneous environments, e.g., on riverbeds,^2^ in which local concentrations of nutrients and oxygen are determined by the local ecosystem; in turn, the local ecosystem may vary over space and time. Although many specific mechanisms used by bacteria that can initiate biofilm formation have been identified,^13^ connecting environmental stimuli to mechanisms that influence when or where biofilms form remains a significant open question.

In the initial stages of biofilm formation, individual bacteria first switch from planktonic to surface associated^14,15^ and subsequently self-assemble into microcolonies. This transition is facilitated by collective surface motility modes, including twitching^16^ and swarming,^17^ that are driven by motility appendages. Twitching requires type-IV pili^16,18^ (TFP), filamentous surface appendages that preferentially locate at the cell poles^19^ and contribute to surface adhesion, signal transduction and pathogenicity.^20,21^ In twitching, bacteria first assemble pilins at one or both poles to extend TFP. The assembly of pilins, including the major pilin PilA and other minor pilins, is driven by a hexameric ATPase (PilB) of the large VirB11 family.^22^ To generate motion, bacteria typically assemble 2 or 3 TFP^23^ and tightly attach their tips to the surface.^24^ To retract the extended TFP, bacteria depolymerise the assembled pilins using a PilB-like ATPase with a typical retraction time of 1–3 s.^23^ The forces exerted by attached TFP during retraction can exceed 100 pN.^25^ By repeated cycles of extension, attachment, and retraction, TFP enable bacteria to move irregularly and twitch on a surface.^26^

Microcolony formation in young biofilms requires normal twitching.^14,27^ Environmental conditions such as iron starvation, however, can disrupt normal twitching of sessile bacteria and thereby prevent biofilm formation.^28^ Because the growth,^29,30^ extension and retraction^31,32^ of TFP are tightly regulated by multiple genes related to the chemotactic and the global second messenger cyclic diguanosine monophosphate (c-di-GMP) signalling pathways,^28,33–36^ bacteria are thought to be able to coordinate TFP-driven motility in response to environmental cues.^37,38^ How bacteria modify their use of TFP-driven motility under different environmental conditions, however, is still poorly understood, especially at the single-cell scale. Bacteria respond to strong spatial chemical gradients over large length scales through chemotaxis. The weak chemical gradients expected to appear in slowly changing natural environments, however, may be insufficient to active chemotaxis signalling pathways that initiate cell response. Hence, bacteria may have evolved other motility strategies that enable them to survive or thrive in slowly changing and locally heterogeneous natural environments. Developing insight into these strategies requires techniques to correlate motility of individual bacteria to the use of motility appendages and is the focus of this study.

Here we identify mechanisms by which *Pseudomonas aeruginosa* bacteria differently deploy TFP in varying environmental conditions. To test the hypothesis that bacteria responsively alter TFP use to generate qualitatively distinct types of motion, we used high-throughput algorithms^39–41^ to track thousands of *P. aeruginosa* moving using TFP on surfaces in varying nutrient conditions and hence identified four characteristic motility types. We related motility types to environmental signals and to TFP use by characterising the subcellular localisation of FimX, a protein that is required for TFP assembly^33^ and is thought to connect environmental signals to twitching.^28^ We found that the subcellular distribution of FimX was asymmetric in bacteria that crawl unidirectionally, consistent with assembly of TFP primarily at the leading pole. By contrast, FimX was distributed symmetrically or was not expressed for bacteria that stall in place. We subsequently applied these techniques to investigate the motility of *P. aeruginosa* in varying nutrient conditions. In nutrient-limited media, bacteria preferentially used unidirectional crawling and FimX was more likely to be asymmetrically distributed within cells. Conversely, in nutrient-supplemented media bacteria preferentially ceased translational motion and FimX was more likely to be symmetrically distributed within cells. To explore the consequences of these changes in single-cell motility behaviours on their ability to inhabit diverse environments, we developed a minimal stochastic model in which the motion of bacteria relies only on the subcellular concentration and localisation of FimX. The model reproduced the quantitative characteristics of each motility type while maintaining the broad distributions seen in experiment. Applying this model, we found that distinct motility types could increase the efficiency of searching or clustering, processes involved in the decision by bacteria to form early-stage biofilms. These findings suggest one way in which individual mechanisms of motility could affect population-scale fitness: by altering single-cell TFP use via polarity of TFP assembly, *P. aeruginosa* can adopt motility mechanisms favourable for the environmental conditions; nonetheless, by maintaining diversity of motility types across the population bacteria can readily exploit local changes in these conditions.

## Results

### *P. aeruginosa* exhibits four TFP-driven motility types on surfaces

**Figure 1:**
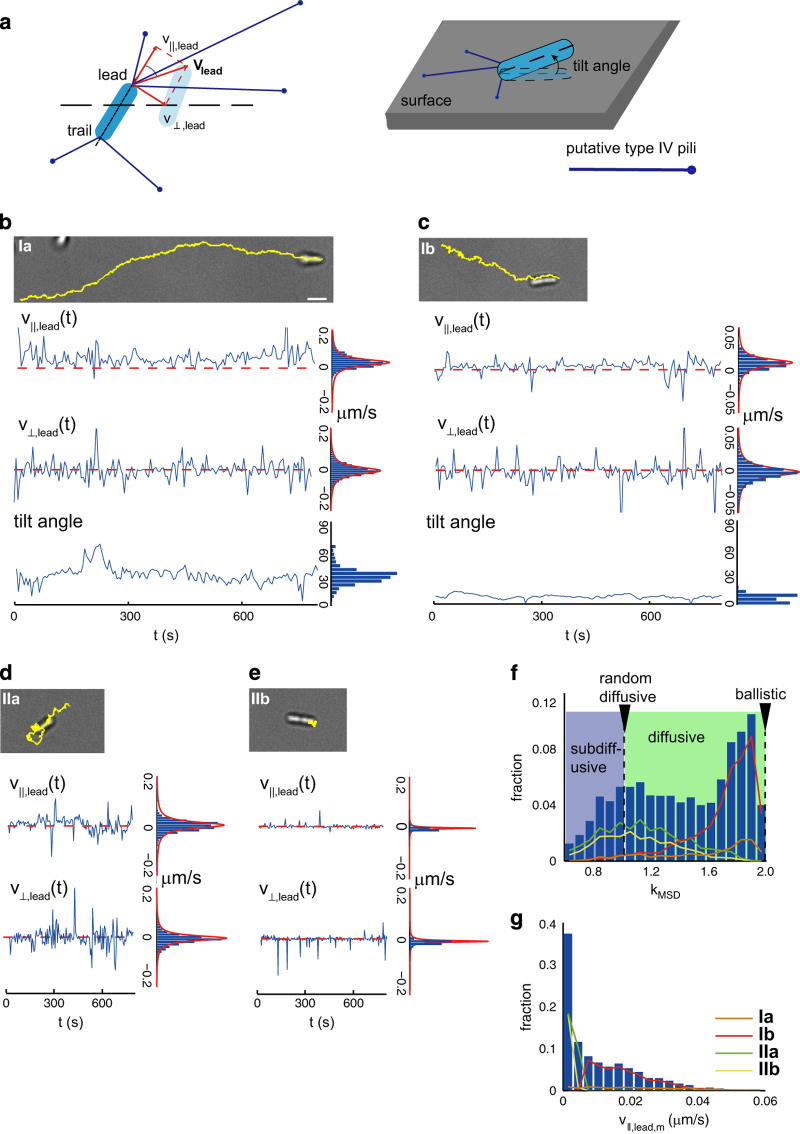
Distinctive twitching motility types in *P. aeruginosa*. (**a**) (left) Schematic illustrating the decomposition of the instantaneous velocity (**v**_lead_(*t*)) at the leading pole into components along (e.g., *v*_||,*lead*_(*t*)) and perpendicular (e.g., *v*_⟂,lead_(*t*)) to the body axis of the bacterium. (right) Schematic illustrating the tilt angle at which the body of the bacterium was inclined relative to the surface. (**b**–**e**) Representative twitching motility types for (**b**) type Ia: unipolar-attached crawling cell; (**c**) type Ib: bipolar-attached crawling cell; (**d**) type IIa: bipolar-attached wiggling cell; (**e**) type IIb: bipolar-attached stalling cell, in which sub-panel represents brightfield micrographs, the components of the instantaneous velocity **v**_lead_(*t*) (*v*_||,lead_(*t*) and *v*_⟂,lead_(*t*)) at the leading pole along and normal to the axis of cell body, tilt angle and their corresponding distributions, respectively. Yellow lines in brightfield micrographs indicate the trajectories of the leading pole. Lines in the histograms of *v*_||,lead_(*t*) and *v*⟂_,lead_(*t*) represent fits to a Cauchy–Lorentz distribution. The scale bar for all micrographs is 2 μm, as shown in **b**. (**f, g**) Histograms of **f** the slope of the mean-squared displacement *k*_MSD_ and (**g**) the median velocity along the body axis of the cell at the leading pole *v*_||,*lead,m*_ generated from ~3500 cells. The blue or green shading in **f** indicates subdiffusive (*k*_MSD_<1) or diffusive region (1⩽*k*_*MSD*_<2), respectively. Coloured lines indicate the contribution from each distinctive motility type.

We first classified the trajectories of flagella-deficient *P. aeruginosa* mutants (Δ*fliM*) from bright-field microscopy movies using a high-resolution two-point tracking assay.^41^ We separately tracked the two poles of each bacterium^41^ and thereby obtained distributions of the velocities of the leading (*v*_lead_) and trailing (*v*_trail_) poles of the bacteria, which were further resolved into components along (e.g., *v*_||,lead_) and perpendicular (e.g., *v*_⟂,lead_) to the body axis of the bacterium, as shown in Figure 1a. All velocity distributions (*p*(*v*_||,lead_) or *p*(*v*_⟂,lead_)) could be fitted to the Cauchy–Lorenz form, *p*(*v*)=*σ*/*π*[(*v*–*v*_m_)^2^+*σ*^2^], as shown in Supplementary Figure S1, which allowed us to extract both the median velocity (*v*_||,lead,m_ or *v*_⟂,lead,m_) and the characteristic full-width at half maximum *σ*. We also estimated the tilt angle *θ* at which the body of the bacterium was inclined relative to the surface (Figure 1a and Supplementary Figure S1).

Using these metrics, we identified four distinct TFP-driven motility types in Δ*fliM* cells. We focused on motion along the axis of the body (i.e.,*v*_||*,*lead_) to emphasise those movements that resulted from coordinated pulling of TFP. Two types that gave rise to net translational motion could be distinguished by the time-averaged tilt angle $\bar{\theta }$: in unipolar crawling (type Ia: $\bar{\theta }>10{}^{\circ}$, Figure 1b and Supplementary Movie S1), the body of the bacterium was tilted away from the surface, and in bipolar crawling (type Ib: $\bar{\theta }⩽10{}^{\circ}$ and *v*_||,lead,m_>0.006 μms^−1^, Figure 1c and Supplementary Movie S2), the body of the bacterium remained close to the surface. Bacteria attached at one pole (type Ia) could switch between unipolar crawling and the previously identified walking:^39^ in this case their tilt angles approached 90° and the persistence length of the trajectory decreased (Supplementary Figure S2a and Supplementary Movie S5). Two additional types did not generate net translational motion, but could nonetheless be distinguished by the width of the velocity distribution (*σ*): in bipolar-attached wiggling (type IIa: $\bar{\theta }⩽10{}^{\circ}$, *v*_||,lead,m_⩽0.006 μms^−1^, and *σ*_||,lead_>0.003 μms^−1^, Figure 1d and Supplementary Movie S3), the bacterium moved back-and-forth around a fixed point of attachment, whereas in bipolar-attached stalling (type IIb: $\bar{\theta }⩽10{}^{\circ}$, *v*_||,lead,m_⩽0.006 μms^−1^, and *σ*_||,lead_⩽0.003 μms^−1^, Figure 1e and Supplementary Movie S4) the bacterium was nearly immobile. Type IIb motility was comparable to that observed for a double-mutant Δ*fliC*Δ*pilA* that lacked motility appendages and was thus expected to be immobile (Supplementary Figure S2b and Supplementary Movie S6), indicating that pulling of TFP did not drive type IIb motion. For the remaining motile types (Ia, Ib, IIa) *v*_⟂,lead,m_~0, indicating that there was no bias in the lateral motion resulting from asymmetric pulling across the long body axis of the bacterium.

To quantify the extent to which each motility type enabled directional motion, we calculated the mean-squared displacement (MSD) for each trajectory and extracted its slope *k*_MSD_ (see Materials and Methods). A value of *k*_MSD_=1.0 corresponded to random diffusive motion, whereas *k*_MSD_=2.0 corresponded to directional ballistic motion. The average value of 〈*k*_MSD_〉 for types that generate net translational motion (type Ia 〈*k*_MSD_〉=1.55±0.34 and type Ib 〈*k*_MSD_〉=1.70±0.24) was larger than that for types in which the centre-of-mass of the bacterium did not move (type IIa 〈*k*_MSD_〉=1.15±0.28 and type IIb 〈*k*_MSD_〉=1.11±0.26; Figure 1f and Supplementary Figure S3). Although the average values were consistent with the net translation of the centre-of-mass in each type, *k*_MSD_ was nonetheless widely distributed for the different motility types (ranges: 0.68 to 1.98 (Ia), 0.60 to 2.0 (Ib), 0.6 to 1.90 (IIa) and 0.61 to 1.85 (IIb)) and thus was not directly correlated with type. Indeed, distributions of *v*_||,lead,m_ and *k*_MSD_ for each of the four motility types showed significant overlap (Figure 1f, g).

Within a representative population of bacteria, we identified all four motility types. Across ~3,500 Δ*fliM* bacteria, the subpopulations exhibiting motility types Ia, Ib, IIa, and IIb contained 6±0.4%, 34±0.9%, 35±0.9% and 25±0.8% of the total population (Supplementary Figure S3). Over the typical duration of a trajectory (approxiamtely 30–60 min, somewhat less than the doubling time of ~80 min), bacteria using motility types Ib, IIa and IIb did not typically switch type. Bacteria employing type Ia motility, however, exhibited a ~75% chance to switch to walking between consecutive frames, which were separated by 0.1 s (Supplementary Movie S5); such rapid switching between motilities was found only for this motility type.

### Expression of FimX is required for motility types driven by pulling of TFP

Inspired by the ‘tug-of-war’ mechanism by which multiple pilus motors cooperate in *Neisseria gonorrhoeae*,^23,42^ we hypothesised that competition between TFP pulling at the leading and trailing poles of the bacterium gives rise to these distinct motility types. In this picture, we expect that asymmetric pulling of TFP at one pole will generate directional translation of the bacterium with a high velocity, whereas symmetric pulling of TFP at both poles will prevent net translation and thus cause the bacterium to oscillate around a fixed position.

Testing this picture required a quantitative metric to gauge TFP use at the poles of the bacterium. We chose not to directly label the TFP with a fluorescent dye for direct imaging, because exogenous labelling by a synthetic dye affects TFP-driven motility. Instead, as an endogeneous label we created a fusion protein consisting of RFP and the protein FimX (PA4959), which localises at one or both poles of *P. aeruginosa* bacteria^33^ and is thought to regulate twitching motility in response to environmental stimuli.^28^ By expressing this fusion protein, we were able to visualise the subcellular distribution of FimX. Mutants lacking this protein (∆*fimX*) expressed normal levels of the protein pilin that composes TFP, but exhibited fewer surface TFP and were thus deficient in twitching motility.^28^ To determine the effect of FimX expression on the four motility types, we characterised the surface motility of ∆*fimX* mutants. Motility types Ia, Ib and IIa completely disappeared in ∆*fimX* mutants and compensation of *fimX* restored them (Supplementary Figure S4). These results confirmed that expression of FimX is required for motility driven by pulling of TFP.

### Motility types reflect differences in the subcellular localisation of FimX

**Figure 2:**
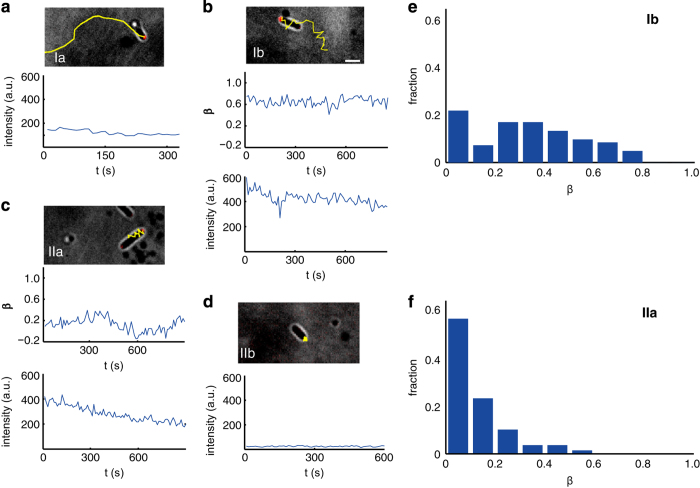
Subcellular localisation of FimX correlates to twitching motility type in *P. aeruginosa*. (**a**–**d**) Representative subcellular localisation of FimX for (**a**) an unipolar-attached crawling cell (type Ia); (**b**) a bipolar-attached crawling cell (type Ib); (**c**) a bipolar-attached wigging cell (type IIa); (**d**) a bipolar-attached stalling cell (type IIb); each panel includes both a combined brightfield+fluorescence image and a corresponding time series of the FimX symmetry parameter *β* and/or fluorescence intensities. Images were created by overlaying brightfield and fluorescence micrographs. (**e**, **f**) Histogram of *β* for cells employing (**e**) type Ib and (**f**) type IIa motility. *β*=0.25 corresponds to a fluorescence intensity at the trailing pole that is 3/5 of that at the leading pole, i.e., *I*_trail_/*I*_lead_=(1−*β*)/(1+*β*)=3/5; similarly, *β*=0.5 corresponds to *I*_trail_/*I*_lead_=(1−*β*)/(1+*β*)=1/3. Cells employing type Ib motility were more likely than those using type IIa motility to exhibit an asymmetric subcellular distribution of FimX.

To correlate the subcellular distribution of FimX to the motility types, we used spinning-disk confocal microscopy to quantify the localisation of red fluorescent protein (RFP)-tagged FimX in individual bacteria. We carefully optimised the imaging protocol (laser power and exposure time) to confirm that the motility of bacteria was unaffected. In motile bacteria (employing type Ia, Ib or IIa motility), FimX was typically asymmetrically (Figure 2b and Supplementary Movie S8) or symmetrically (Figure 2c and Supplementary Movie S9) localised at the cell poles. To quantify this asymmetry we defined a dimensionless symmetry parameter *β*≡(*I*_lead_−*I*_trail_)/(*I*_lead_+*I*_trail_), where *I*_lead_ and *I*_trail_ were the local fluorescence intensities of RFP-tagged FimX at the leading and trailing poles, respectively, and 0⩽|*β*|⩽1. A value of |*β*|=0 corresponded to a symmetric distribution of FimX at both poles, whereas |*β*|=1 corresponded to FimX localised at one of the two poles. We explicitly assumed that the fluorescence intensity measured by the confocal microscope was linearly proportional to the concentration of FimX at the poles, i.e., *β*=(*c*_lead_−*c*_trail_)/(*c*_lead_+*c*_trail_).

Each mobile type exhibited a characteristic range of values of the symmetry parameter *β*. For bacteria using type Ib motility (bipolar crawling) a significant fraction of cells exhibited *β*>0 (Figure 2b and Supplementary Movie S8), i.e., FimX was localised at the leading pole. Similarly, bacteria using type Ia motility (unipolar-attached crawling) also tended to exhibit asymmetric distributions of FimX (Figure 2a and Supplementary Movie S7). Conversely, for bacteria exhibiting the non-translational type IIa motility (bipolar-attached wiggling) *β* approached zero (Figure 2c and Supplementary Movie S9), i.e., FimX was distributed symmetrically at the two poles. Immobile bacteria (those employing type IIb motility) exhibited nearly zero fluorescence intensity (Figure 2d and Supplementary Movie S10), confirming that expression of FimX was required for surface motility. Distributions of *β* for translational type Ib (Figure 2e) and non-translational type IIa (Figure 2f) indicated that a greater percentage of cells exhibited asymmetric subcellular distributions of FimX when undergoing directional motion. Furthermore, *β* was positively correlated with *v*_||,lead,m_ (correlation coefficient: 0.92) and *k*_MSD_ (correlation coefficient: 0.83). This finding intriguingly hints that increasing asymmetry in the expression of FimX allows the bacterium to move more rapidly and directionally on the surface.

### Nutrient conditions affect the subpopulations of TFP-driven motility types

**Figure 3:**
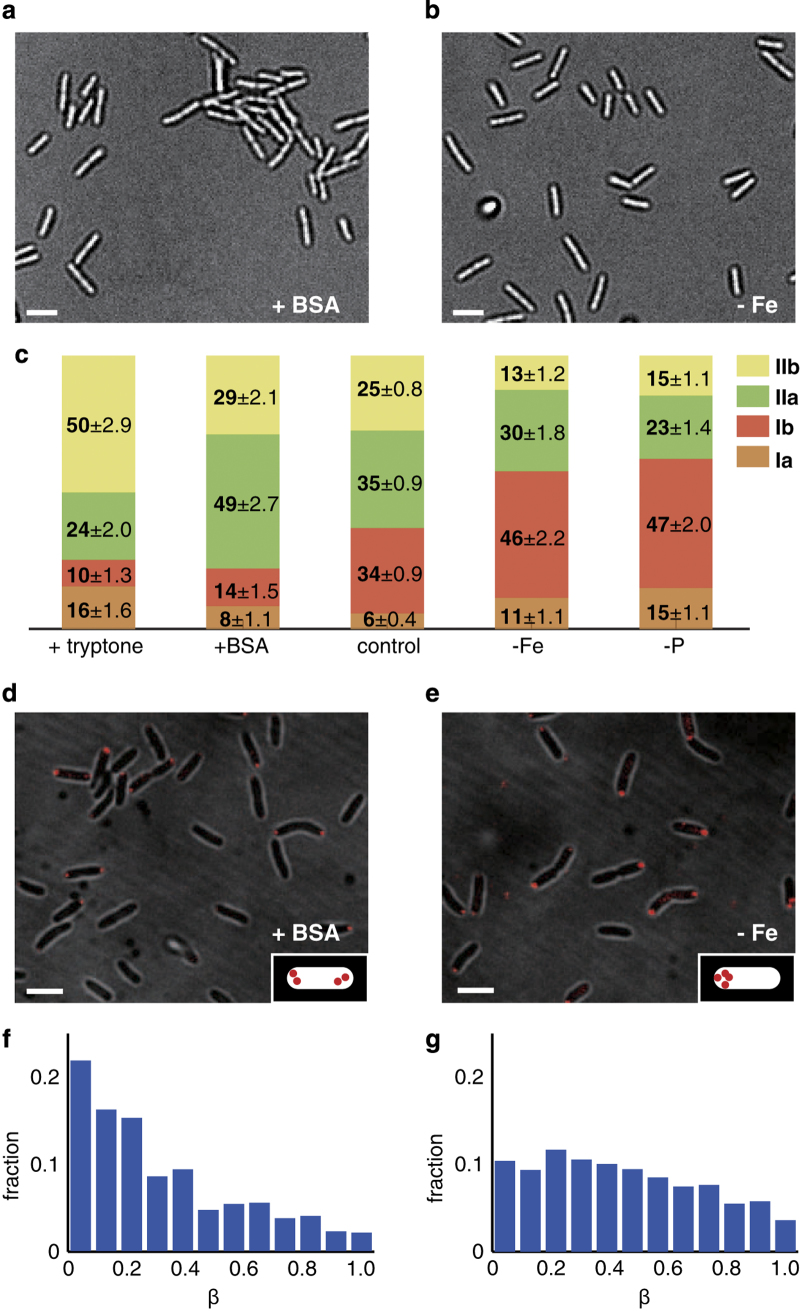
Twitching motility types in *P. aeruginosa* depend on nutrient availability. (**a**, **b**) Brighfield micrographs show that cells preferentially form clusters in **a** nutrient-supplemented (+0.1% BSA) or disperse in **b** nutrient-limited condition (starvation of iron). (**c**) Motility types of *P. aeruginosa* under nutrient-supplemented (addition of tryptone or BSA) or nutrient-limited (starvation of iron or phosphate) conditions. (**d**, **e**) Brightfield+fluorescence images show expression and subcellular distribution of FimX in nutrient-supplemented (+0.1% BSA, **d**) or nutrient-limited condition (starvation of iron, **e**). Images were created by overlaying brightfield and fluorescent micrographs. (**f**, **g**) Histogram of the symmetry parameter *β* in nutrient-supplemented (+0.1% BSA, **f**) or nutrient-limited condition (starvation of iron, **g**). Scale bar for all images is 2 μm.

Earlier studies showed that distinctive nutrient conditions affect the twitching zones^28^ that can be measured using a classical twitching motility assay, suggesting that bacteria can deploy their TFP to adapt to a broad range of nutrient conditions. To discover the general principles that determine how bacteria use their TFP to respond to nutrient availability, we added or subtracted nutrients from the minimal (FAB) medium typically used for biofilm experiments on *P. aeruginosa* and imaged bacteria moving on the surface. We chose to add or subtract nutrients rather than varying the concentration of a given nutrient to maximise the effects of nutrient change. When the medium was supplemented with an additional nutrient such as bovine serum albumin (BSA), bacteria formed small and immobile clusters on the surface (Figure 3a). Conversely, when the medium was starved of a critical nutrient such as iron, bacteria remained dispersed and mobile (Figure 3b). We conjectured that differences in spatial distribution reflect underlying differences in the types of motility favoured by bacteria under varying nutrient conditions. We thus characterized the motility types of bacteria under a variety of nutrient-supplemented (addition of tryptone or BSA) or nutrient-limited (starvation of iron or phosphate) conditions (Figure 3c and Supplementary Figure S5). In nutrient-supplemented conditions, relative to the control experiment bacteria preferentially deployed the type IIa and/or type IIb motilities that did not generate net translation. Conversely, in nutrient-limited conditions, bacteria preferentially deployed the type Ia and/or type Ib motilities that enabled them to translate across the surface. Furthermore, the distribution of FimX tended to be more asymmetric under nutrient-limited conditions (Figure 3d) and more symmetric under nutrient-supplemented conditions (Figure 3e), in general accord with these motility types, although the asymmetry parameter *β* in both nutrient-supplemented (Figure 3f) and nutrient-limited (Figure 3g) conditions was broadly distributed.

Although this finding is consistent with the idea that *P. aeruginosa* can differently deploy TFP to adapt to nutrient availability by regulating the subcellular average concentration and asymmetry of FimX, changing nutrient conditions may also alter other factors that would cause bacteria to differently use TFP-driven motilities. For example, altered nutrient conditions could affect (i) the metabolic rate of bacteria and/or (ii) the production of rhamnolipid biosurfactants (under iron-limited conditions), which can attenuate the interactions between the cell body and the surface.^5^ To rule out (i), we determined whether the metabolic rate in single cells was correlated with use of TFP-driven motilities in different nutrient conditions. The growth of cells was almost uncorrelated with *v*_||,lead,m_ (Supplementary Figure S6a, correlation coefficient: 0.16) in all tested nutrient conditions and hence did not drive the changes in motility observed here. To rule out (ii), we investigated whether a double-mutant (Δ*rhlA*Δ*fliM*) that was deficient in producing rhamnolipids^43^ could preferentially deploy type Ia and/or type Ib motilities when starved of iron. The mutant Δ*rhlA*Δ*fliM* still preferentially deployed Ib motility in iron-limited conditions (Supplementary Figure S6b); we note, however, that type Ia motility almost disappeared in the Δ*rhlA*Δ*fliM* mutant, which is consistent with the idea that rhamnolipids reduce the interactions between the cell body and the surface.

### Average concentration and subcellular distribution of FimX describes the TFP-driven motility of *P. aeruginosa*

**Figure 4:**
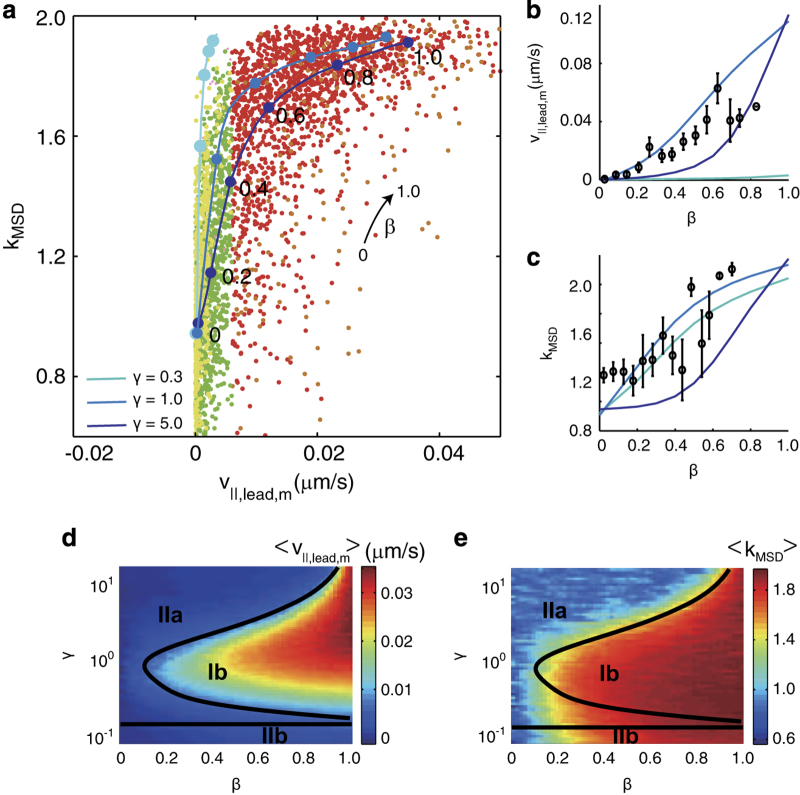
Minimal stochastic model reproduces the motility types from the subcellular average concentration and asymmetry of FimX. (**a**) Slope of the mean-squared displacement *k*_*MSD*_ as a function of median velocity along the cell body at the leading pole *v*_||,lead,m_. Yellow, green, red and brown symbols represent the cells using type Ia, Ib, IIa and IIb motilities, respectively. Solid lines represent simulation curves generated using the minimal stochastic model with varying values of *β* and *γ*. Experimentally-generated (**b**) 〈*v*_||,lead,m_〉 and (**c**) 〈*k*_*MSD*_〉 as a function of *β*. Vertical lines indicate standard error and solid curves indicate simulation results generated from different values of *γ*, as in (**a**). (**d**) 〈*v*_||,lead,m_〉 and (**e**) 〈*k*_MSD_〉 as a function of *β* and *γ*, obtained from simulation; colours indicate magnitudes of 〈*v*_||,lead,m_〉 and 〈*k*_MSD_〉 as indicated. The solid lines in **d** and **e** represent the boundaries of the distinctive motility types.

To further explore how the combination of subcellular average concentration and asymmetry of FimX produces different types of TFP-driven motility, we built a minimal Monte Carlo simulation to relate subcellular localisation of FimX to motility. In our simulation model, the velocity and orientation of the bacterium were controlled by the probability of retraction of TFP at the leading (*p*_lead_) or trailing (*p*_trail_) pole, which in turn were positively correlated with the local concentration of FimX. (Additional details are given in Supplementary Methods and in Supplementary Figure S7). We assumed that the retraction probability at the leading or trailing poles was given by *p*_lead_=1–exp(−*c*_lead_/c*) or *p*_trail_=1–exp(−*c*_trail_/c*), respectively, where c* is the characteristic concentration of FimX that enables assembly of TFP. We defined a dimensionless concentration *γ*=*c*_*t*_/c*, where *c*_*t*_ is the average subcellular concentration of FimX. Simulated trajectories therefore depended only upon the two dimensionless parameters *β* and *γ*; we explored the range 0.1⩽*γ*⩽20, where the limits *γ*«1 and *γ*»1, respectively, correspond to non-expression or overexpression of FimX. Although our two-dimensional model cannot simulate type Ia motility because we do not account for the tilt angle, we expect that type Ia motility should be captured by the limit *β*→1 if the crawling-to-walking transition is neglected. Our model reproduced the positive correlation between *v*_||,lead,m_ and *k*_MSD_ found in our experimental data for intermediate values of *γ*, as shown by the solid lines in Figure 4a. A lower value of *γ*=0.3 was consistent with bacteria using type IIb motility, and a higher value of *γ*>1.0 and a nonzero value of *β*>0.2 was consistent with bacteria using type Ia or type Ib motility. As an additional test of our model, we examined the dependence of *v*_||,lead,m_ and *k*_MSD_ on the asymmetry parameter *β*. Generally, as *β* was increased, so that the distribution of FimX became more asymmetric, both *v*_||,lead,m_ and *k*_MSD_ increased (Figures 4b and c). Moreover, the intermediate values of *γ* that described the correlation between *v*_||,lead,m_ and *k*_MSD_ in Figure 4a also captured the dependence of *v*_||,lead,m_ and *k*_MSD_ on *β*.

To explore how FimX concentration and asymmetry affected the distinctive motility types in our model, we classified the motility types of all simulated trajectories using the criteria established earlier. We found, generally, that non-expression of FimX (*γ*<0.3) resulted in type IIb motility. By contrast, type Ib and IIa motilities depended non-monotonically on both *β* and *γ*, although bacteria in which FimX was symmetrically localised at the poles (*β*→0) generally used type IIa motility in agreement with our experimental findings (Figure 2c, f). Bacteria using type Ib motility typically exhibited larger average values of *v*_||,lead,m_ and *k*_MSD_, in good agreement with the experimental results (Figure 4d, e). Moreover, because multiple distinct combinations of *β* and *γ* could give rise to each type of motility, our minimal model could generate the wide variation in *v*_||,lead,m_ and *k*_MSD_ seen in the experimental data. The good agreement between experimental and simulation data suggests that our minimal model, which includes only the average subcellular concentration and extent of localisation of FimX, can generate all of the features of TFP-driven motility used by *P. aeruginosa* to move on surfaces.

### Searching and clustering efficiencies are optimised for different motility types

Our experiments suggest that bacteria vary their motility strategies in different nutrient conditions. To connect these different strategies to decisions made by bacteria during early biofilm formation, we noted that increasing velocity along the direction of motion (*v*_||,lead,m_), typically associated with type Ia or type Ib motility, was positively correlated with increasing linearity or directionality of the trajectory (i.e., an increase in *k*_MSD_), as shown in Figure 4a. This finding is consistent with the idea that bacteria can deploy TFP to move rapidly and cover linear distance.^39,40^ We therefore considered ways in which these distinctive motility types could impart advantages to individual bacteria and/or to the survival of a bacterial population.

**Figure 5:**
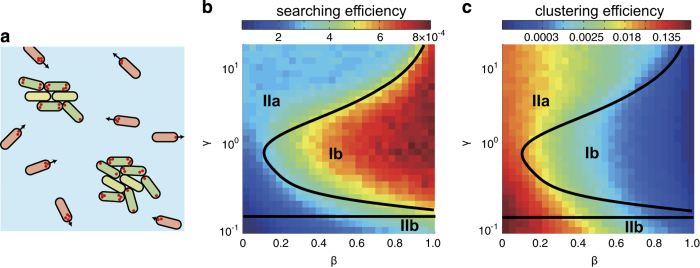
Different roles are enabled by distinctive twitching motility types. (**a**) Schematic showing that motility types Ib and IIa/IIb impart different capabilities for searching or clustering. (**b**) Searching and (**c**) clustering efficiency as a function of *β* and *γ*, obtained from simulation; colours indicate the magnitudes of the efficiencies as indicated. The solid lines represent the boundaries of the distinctive motility types.

First, we conjectured that the ability to translate large distances could give bacteria that were searching for desirable targets, such as nutrients, a significant advantage in rapidly and efficiently locating them. This idea is motivated by earlier studies of spatiotemporally varying environments, such as marine habitats, in which rapid motility allows bacteria to exploit transient microscale patches.^44^ Second, we considered potential advantages of non-translational motility. In a competitive landscape, rapid proliferation in a favourable niche can allow bacteria to outgrow competitors;^45^ in the context of early biofilm formation, bacteria must be able to stop moving once a niche is identified but also cluster tightly to communicate^46^ and share beneficial goods.^47^ Hence, we conjectured that the ability to arrest translational motion by adopting non-translational motility types would correlate with increased clustering efficiency. Furthermore, we proposed that the distinctive TFP-motility types as well as their diversities may afford the bacteria robust adaptability in surface motility (Figure 5a). We applied the minimal simulation model to test each of these conjectures.

First, to quantify the searching efficiency we randomly distributed target sites across a surface at a fixed density. For each bacterium we calculated the first successful time searching of a target site *t*_*s*_ and thereby defined for each trajectory a target searching efficiency *η*_*s*_(*β*, *γ*)=Δ*t*/〈*t*_*s*_〉 as the ratio between the simulation time step (Δ*t*) and the average searching time *t*_*s*_. Type Ib motility resulted in a higher searching efficiency than the other motility types (Figure 5b). Second, we defined a clustering efficiency *η*_*c*_(*β*, *γ*)=*N*_*c*_/*N* as the average number of bacteria in clusters after a fixed time period. Type IIa and IIb motilities typically exhibited higher clustering efficiencies than type Ib motility (Figure 5c), consistent with the propensity for bacteria to remain dispersed and mobile under nutrient-limited conditions (Figure 3b) and to cluster and form microcolonies under nutrient-supplemented conditions (Figure 3a). Clustering in our simulation was driving solely by motility; the ability to sense gradients (i.e., chemotaxis) was not included and was not required to optimise clustering efficiency. Finally, searching and clustering efficiencies were nearly disjoint in the two-dimensional (*β*, *γ*) phase space, indicating that the translational and non-translational TFP-driven motility types possess complementary functions. This result suggests that bacteria may use different motility types to promote or suppress biofilm formation, depending on the environmental conditions.

## Discussion

*P. aeruginosa* employs different surface motility strategies in varying nutrient conditions: in nutrient-limited conditions bacteria preferentially use translational motility types, whereas under nutrient-supplemented conditions bacteria preferentially arrest using non-translational motility types. These phenotypes, in turn, are correlated with asymmetric or symmetric expression of FimX, a protein involved in the assembly of TFP that contains functional domains implicated in environmental response. In nutrient-limited conditions, *P. aeruginosa* are more likely to asymmetrically deploy TFP at one pole to crawl in a straight line, whereas in nutrient-supplemented conditions *P. aeruginosa* are more likely to symmetrically pull with TFP or not assemble them to arrest at a single surface location. In this fashion, *P. aeruginosa* can optimise its appendage-driven motility to translate or to arrest, depending on nutrient availability. As one consequence, by strategically using the different motility types bacteria are able to identify and select a suitable place to form microcolonies, depending on whether the nutrient conditions are favourable or unfavourable for growth. Note that this simple strategy differs from the adaptive strategy driven by chemotaxis; namely, whether bacteria decide to translate or arrest depends on nutrient availability rather than nutrient gradients. The ability to change motility in response to local conditions (rather than gradients) may enhance the tolerance of bacteria for unexpected fluctuations of nutrient availability in environmental settings. Slow variations in nutrient concentration over time, for example, may not generate a perceptible spatial gradient, and hence bacteria that employ only the sensing pathway of chemotaxis may not be able to respond to or adapt to these conditions.

Our minimal model indicates one possible origin of the diversity of TFP-driven motility behaviours observed in experiments. Various combinations of the average and local subcellular concentrations of FimX can generate motility types Ia/b and IIa/b as well as the broad diversity found in Ia, Ib and IIa. Redundancy in TFP-driven motility behaviours may help to protect a bacterial population in a spatiotemporally fluctuating environment; as one example, nutrient availabilities in a riverbed ecosystem slowly changes with the seasons.^2^ We suggest that the multiple motility strategies available to each bacterium may generate an ‘insurance effect’^48^ or ‘bet-hedging’^49^ strategy and thereby increase the scope of conditions in which the community can thrive. In addition, because appendage-driven motility is connected to biofilm structure,^15^ the heterogeneous structure of biofilms may originate from this diversity of motility types and appear surprisingly early during formation.

Our results have additional implications for biofilm formation. First, we found that *P. aeruginosa* could pull in opposite directions with TFP to arrest on a surface. These arrested bacteria, employing type IIa motility, express FimX at each pole and actively retract the assembled TFP there without generating directional motion. This mechanism differs from the tug-of-war proposed for twitching in *N. gonorrhoeae*^23,42^ by the absence of net translation. Instead, this motility type complements other physical mechanisms known to halt cell motility and promote microcolony formation on a surface, such as expression of extracellular polysaccharide glues.^50^ Although extracellular polysaccharides can guide TFP-driven motility during early biofilm formation,^51,52^ an intriguing question raised by our results is whether the retraction of TFP at opposite poles (i.e., deployment of motility appendages) can cue *P. aeruginosa* to produce EPS and thereby promote microcolony formation.

Second, our results provide new insight into how *P. aeruginosa* responds to environmental cues. To respond to environmental changes by forming biofilms requires sensing systems. In swimming bacteria such as *E. coli*, surface sensing prior to biofilm formation is controlled in part by hindering flagellar rotation^53–55^ related to cyclic-di-GMP signalling;^56,57^ likewise, chemical sensing is dominated by the flagellar switch protein^58^ that is controlled by the chemotactic system.^59^ Surface-motile bacteria can also exhibit coupling between sensing and motility: for example, the slow directional switching in *Myxococcus xanthus* gliding, which is driven by assembly of TFP at the trailing pole, is controlled by the chemotaxis motility system *frz.*^60^ In *M. xanthus* localisation of TFP components at the pole drives symmetry breaking, as is also suggested by our results. *M. xanthus*, however, assembles TFP only at a single pole, whereas by contrast *P. aeruginosa* can assemble and retract TFP at one or both poles.^26^ These comparisons suggest that sensing systems have a role in *P. aeruginosa*’s transition from motile to sessile. The correlation between motility types, FimX expression, and nutrient conditions shown here suggests that FimX may participate in the pathway that allows *P. aeruginosa* to modify its motility and thereby respond to nutrient addition or starvation.

In *Pseudomonas* and other bacteria species, synthesis of the intracellular signalling molecule c-di-GMP coincides with biofilm formation.^61^ Among other targets, c-di-GMP promotes production of EPS and inhibits motility in response to environmental stimuli.^62^ How c-di-GMP, which controls a large number of protein targets, achieves signalling specificity in a noisy environment remains poorly understood. Because FimX is controlled by c-di-GMP, our results connecting subcellular localisation of FimX to motility type support one postulated mechanism, subcellular sequestration and/or localisation of c-di-GMP.^63^ Our results therefore suggest that single-cell motility coupled to local protein expression may provide a visual measure by which to quantify the onset of biofilm formation.

## Materials and Methods

### Growth conditions and strains

Five mutants of *P*. *aeruginosa*, Δ*fliM*, Δ*rhlA*Δ*fliM*, Δ*fliC*Δ*pilA*, Δ*fimX* and Δ*fimX*pDimer2-FimX, were used in this study, as shown in Supplementary Table S1. Strains were grown on LB agar plates at 37 °C for 24 h. Monoclonal colonies were inoculated into a 5-ml culturing tube containing 1 ml of minimal medium (FAB) and then grown in a shaker at 37 °C, in which 30 mM glutamate was added as the carbon source. Finally, bacterial cultures were collected at exponential phase, as monitored by their optical densities at 600 nm (OD_600_≈0.8). The resultant culture (20 μl) was further diluted 50 times in 1 ml of fresh FAB medium before injection into the flow cell. Details for construction of Δ*fimX* and Δ*fimX*pDimer2-FimX are given in Supplementary Methods.

### Flow cell experiments in different nutrient conditions and tracking of single cells

Flow cells (Denmark Technical University) were prepared and sterilised using a standard protocol.^64^ Bacterial cultures were injected into flow cells and allowed to rest for 15 min to allow bacteria to attach to the coverslip, after which unattached cells were washed out. Surface-attached cells were subsequently cultured at 30.0±0.1 °C by flowing various FAB media at a constant flow rate (3.0 ml h^−1^); media used included standard FAB (minimal condition); FAB with 90% phosphorus or 100% iron removed; and FAB supplemented with 0.1% (wt) BSA or 5% (wt) tryptone, into each of which 0.6 mM glutamate was added as the carbon source. The combination of a minimal medium (FAB) and a limited carbon source (0.6 mM) has been shown to be an optimised nutrient condition for initiating the formation of *P. aeruginosa* biofilms.^64^ The surface motility of the cells in the first six hours after attachment were imaged using an inverted microscope (Olympus IX81) equipped with a 100× oil objective. One movie, typically containing 20,000 bright-field 16-bit grayscale images of dimension 1800 pixels×1800 pixels, was acquired at a frame rate of 10 fps using a sCMOS camera (Andor Neo). These experiments were repeated multiple times for each identical condition, so that over 6,200 movies (1,600,000 images) were acquired and analysed in total. Grayscale images were first converted to binary images for the detection of single cells using a standard image-processing algorithm that was coded in MATLAB. The *x*–*y* coordinates of the centroids of cell were determined in each frame and subsequently linked into trajectories using a particle-tracking algorithm.^41^ Tracking and analysis of the expression and subcellular localisation of FimX in *P*. *aeruginosa* are given in Supplementary Methods.

### Two-point tracking, analysis and simulations of single trajectories

Single bacterial trajectories were further analysed using a two-point tracking algorithm.^41^ The trajectories of the leading and trailing pole (**r**_lead_(*t*) or **r**_trail_(*t*)) were first denoised using a Daubechies wavelet; subsequently the instantaneous velocities (**v**_lead_(*t*) or **v**_trail_(*t*)) were calculated as Δ**r**_lead_(*t*)/Δ*t* or Δ**r**_trail_(*t*)/Δ*t*. **v**_lead_(*t*) was further decomposed into two components (*v*_||,lead_(*t*) or *v*_⟂,lead_(*t*)) along or normal to the major axis of cell body, as shown in Supplementary Figure S1d. The distribution (*p*(*v*_||,lead_ or *p*(*v*_⟂,lead_)) of *v*_||,lead_(*t*) or *v*_⟂,lead_(*t*) was fitted using a Cauchy–Lorentz distribution *p*(*v*)=*σ*/*π*[(*v*−*v*_m_)^2^+*σ*^2^], where *v*_m_ is the median value of velocity and *σ* is the the full-width half maximum (FWHM). The tilt angle (*θ*(*t*)) in the trajectory was estimated by calculating the arccosine of (|**r**_lead_(*t*)–**r**_trail_(*t*)|–*w*)/(*l*(*t*)–*w*), where *w* is the bacterial width, which is nearly independent of time, and *l*(*t*) is the length of the bacterium, which is obtained by linear regression of the time series of projected length (|**r**_lead_(*t*)–**r**_trail_(*t*)|). The mean-square displacement (MSD) of **r**_lead_(*t*) was calculated as $1/T\int_{0}^{T}{\left[r_{\mathrm{lead}}\left(t\right)-r_{\mathrm{lead}}\left(t+\tau \right)\right]}^{2}dt$, where *T* is the duration of trajectory and *τ* is the time delay. The slope of MSD (*k*_MSD_) was obtained from a linear fit of log(MSD) as a function of log(*τ*), i.e., log(MSD)∝*k*_MSD_log(*τ*). The analysis methods are shown in Supplementary Figure S1. Distinctive TFP-driven motility types were automatically classified using four criteria: Type Ia ($\bar{\theta }>10^{o}$); Type Ib ($\bar{\theta }⩽10^{o}$ and *v*_||,lead,m_>0.006 μms^−1^); Type IIa ($\bar{\theta }⩽10^{o}$, *v*_||,lead,m_⩽0.006 μms^−1^ and *σ*_||,lead_>0.003 μms^−1^); and Type IIb ($\bar{\theta }⩽10^{o}$, *v*_||,lead,m_⩽0.006 μms^−1^ and *σ*_||*,lead*_⩽0.003 μms^−1^). These cutoffs were chosen based on the tolerances of the experimental data. The cutoffs for the time-averaged tilt angle $\left(\bar{\theta }=10^{o}\right)$ and FWHM of the leading pole velocity distribution (*σ*_||,lead_=0.003 μms^−1^) were determined as the experimental tolerance for an immobile double-mutant Δ*fliC*Δ*pilA* strain; explicitly, for that strain we measured $\bar{\theta }⩽5^{o}$ and *σ*_||*,lead*_⩽0.0015 μms^−1^. Finally, the cutoff for the leading-pole velocity (*v*_||,lead,m_=0.006 μms^−1^) was chosen as the minimum nontrivial displacement of the bacterial centre-of-mass that could be resolved over a characteristic time for a trajectory (~10 min). Details on simulations of single trajectories and the method of evaluating the searching/clustering efficiencies are described in Supplementary Methods.
