# Supplementary material for: Bacteria differently deploy type-IV pili on surfaces to adapt to nutrient availability
Source: NPJ Biofilms Microbiomes. 2016 Feb 24;2:15029–. doi: 10.1038/npjbiofilms.2015.29 (PMC5515259; doi:10.1038/npjbiofilms.2015.29)
Supplement: Supplementary Information [file npjbiofilms201529-s1.pdf]

Supplementary Information

Bacteria differently deploy type-IV pili on surfaces to adapt to nutrient availability

Lei Ni[1,+], Shuai Yang[1,+], Rongrong Zhang[1], Zhenyu Jin[1], Hao Chen[2], Jacinta C. Conrad[3,*] and Fan Jin[1,*]

[1] Hefei National Laboratory for Physical Sciences at the Microscale, Department of Chemical Physics, Department of Polymer Science and Engineering, CAS Key Laboratory of Soft Matter Chemistry, University of Science and Technology of China, Hefei 230026, P. R. China;

[2]Coordination Chemistry Institute and the State Key Laboratory of Coordination Chemistry, School of Chemistry and Chemical Engineering, Nanjing University, Nanjing 210093, P. R. China;

[3]Chemical & Biomolecular Engineering Department, University of Houston, TX 77204, USA.

[+]L.N. and S.Y. contributed equally to this work.

*To whom correspondence should be addressed.

E-mail: fjinustc@ustc.edu.cn; jcconrad@uh.edu

Contents

**Supplementary Figure 1 to 7**	**2 -- 8**
**Supplementary Table 1**	**9**
**Supplementary Methods**	**10--14**
**Supplementary References**	**15**

Supplementary Figure 1

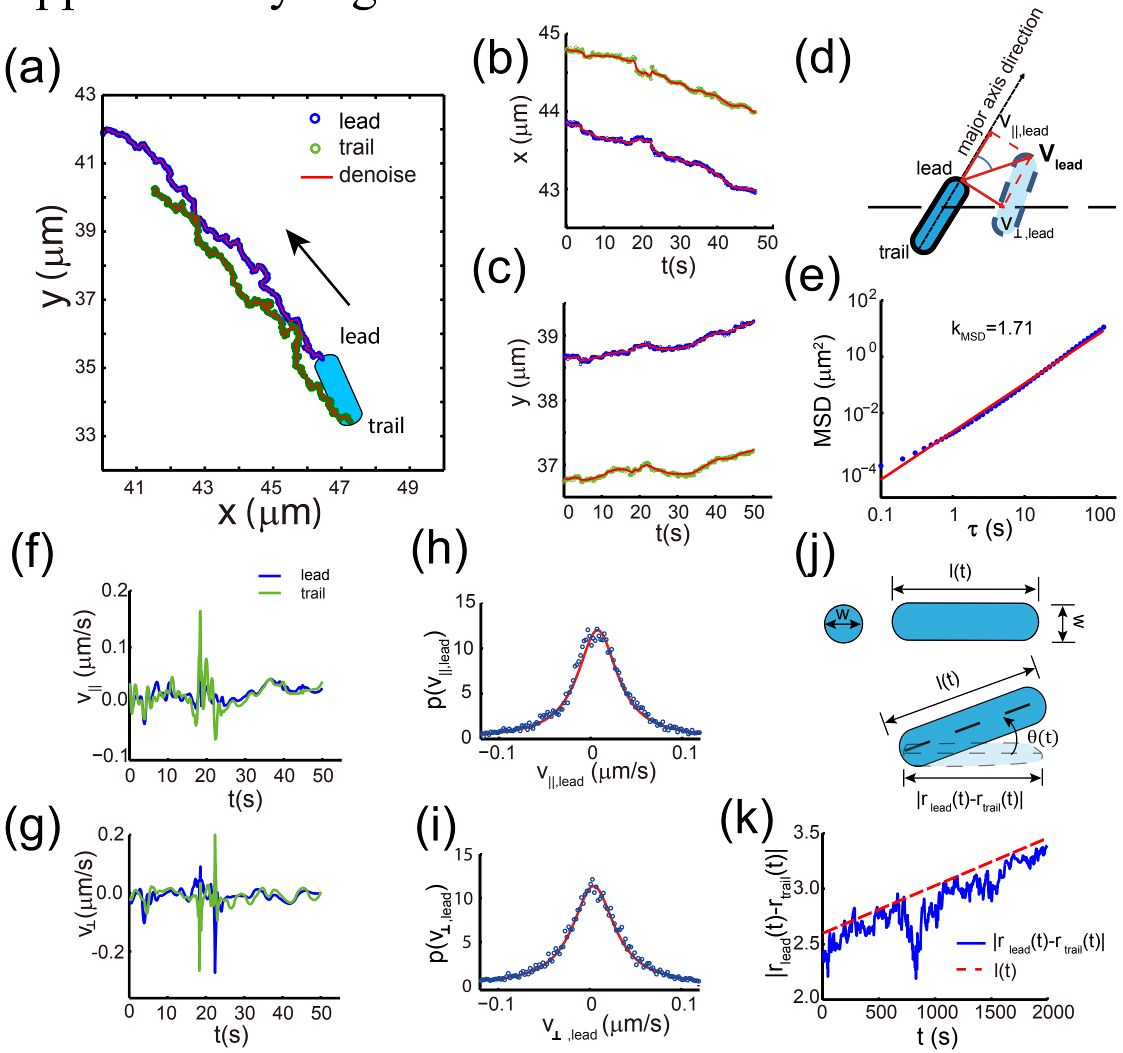

Supplementary Figure 1. Two-point tracking and analysis of single trajectories. (*a* to *c*) Representative two-point tracking of a bipolar attached crawling cell, showing (*a*) trajectories of the leading and trailing poles and the (*b*) *x*-positions and (*c*) *y*-positions as a function of time. Blue and green symbols indicate the *xy*-positions of leading or trailing pole, respectively; red lines indicate the denoised trajectories. (*d*) Schematic showing the decomposition of the instantaneous velocity ($\mathbf{v}_{\text{lead}}(t)$) at the leading pole into components along (*e.g.* $v_{\parallel,\text{lead}}(t)$) and perpendicular (*e.g.*$v_{\perp,\text{lead}}(t)$) to the body axis of the bacterium. (*e*) Mean-square displacement (MSD) as function of τ, where τ is the time delay. The red line shows a linear fit of MSD (τ) in a double logarithmic plot, where k_{MSD} is the slope obtained from the fit. (*f* to *g*) Representative profiles of (*f*) $v_{\parallel,\text{lead}}(t)$, $v_{\parallel,\text{trail}}(t)$ and (G) $v_{\perp,\text{lead}}(t), v_{\perp,\text{trial}}(t)$ as a function of time. (*h* to *i*) Representative distributions of (*h*) $v_{\parallel,\text{lead}}(t)$ and (*i*) $v_{\perp,\text{lead}}(t)$. Red lines in the distributions of $v_{\parallel,\text{lead}}(t)$ and (*i*) $v_{\perp,\text{lead}}(t)$ indicate fits to a Cauchy-Lorentz distribution. (*j*) Schematic showing the tilt angle $\theta(t)$ at which the body of the bacterium was inclined relative to the surface, where $l(t)$ or w is the length or the width of the cell and $|\mathbf{r}_{lead}(t) - \mathbf{r}_{trail}(t)|$ is the projected length. (*k*) Linear regression of representative $|\mathbf{r}_{lead}(t) - \mathbf{r}_{trail}(t)|$. The red dashed line indicates the regression line.

Supplementary Figure 2

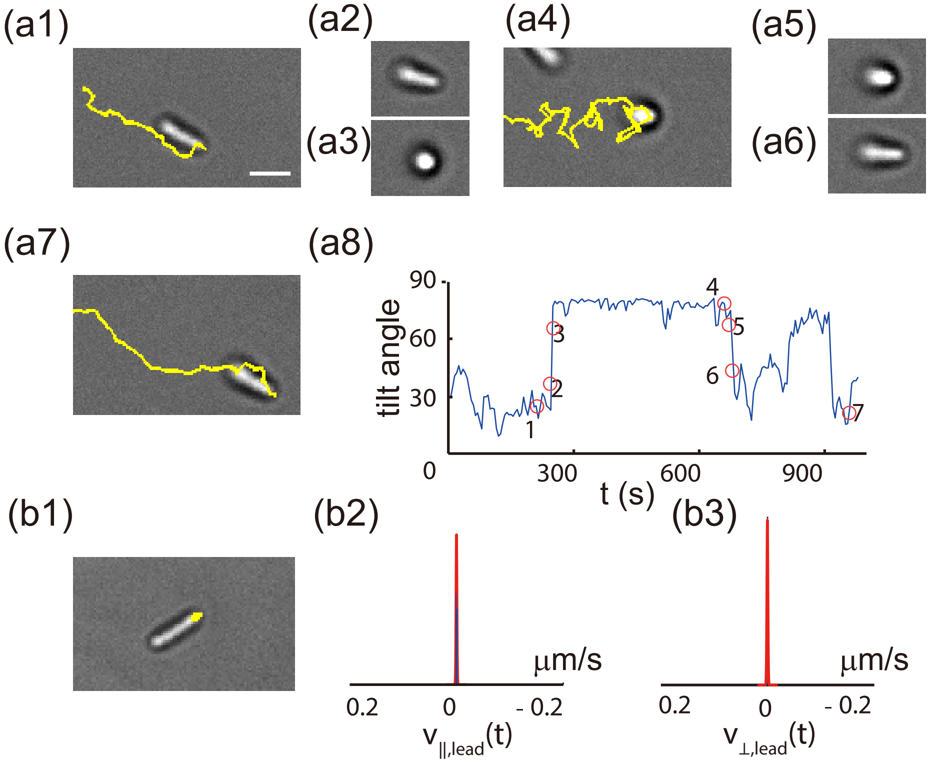

Supplementary Figure 2. Type Ia motility and non-motile phenotype in *P. aeruginosa*. (*a1* to *a8*) Representative brightfield micrographs and time series of tilt angle $\theta(t)$ for (*a1*) unipolar-attached crawling (Type Ia), (*a2*, *a3*) switching to walking, (*a4*) walking, (*a5*, *a6*) switching to Type Ia, (*a7*) Type Ia and (*a8*) $\theta(t)$. (*b1* to *b3*) Representative brightfield micrographs and velocity distributions for a Δ*fliC*Δ*pilA* cell. Yellow lines in brightfield micrographs indicate the trajectories of the leading pole. The scale bar for all micrographs is 2 μm, as shown in (*a1*).

Supplementary Figure 3

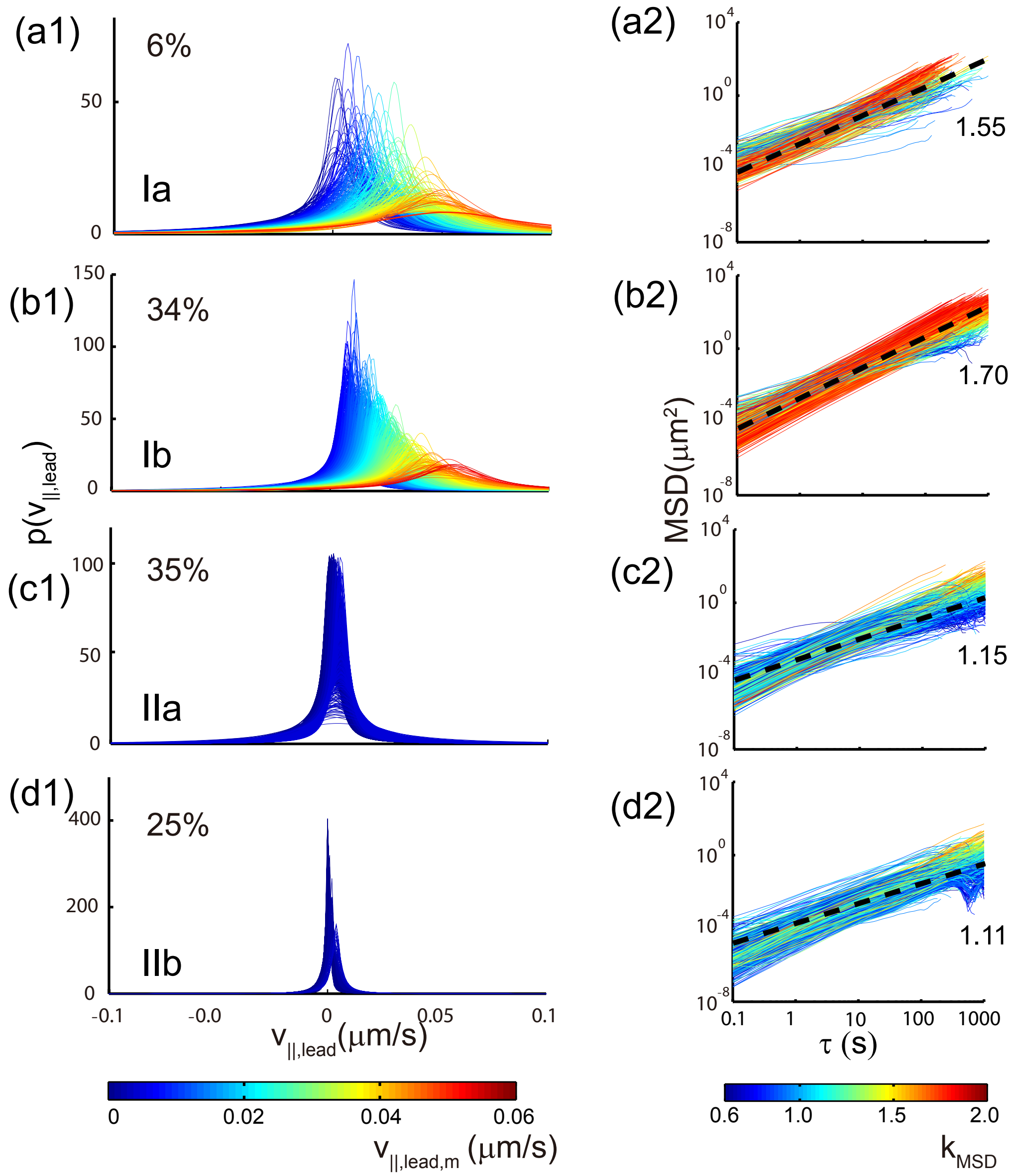

Supplementary Figure 3. Velocity distributions and mean-square displacements in 3500 Δ*fliM* cells. (*a1*, *a2*) Type Ia: unipolar-attached crawling cell. (*b1*, *b2*) Type Ib: bipolar-attached crawling cell. (*c1*, *c2*) Type IIa: bipolar-attached wiggling cell. (*d1*, *d2*) Type IIb: bipolar-attached stalling cell. Colors indicate magnitudes of $v_{\parallel,\mathrm{lead,m}}$ and k_{MSD} as indicated.

Supplementary Figure 4

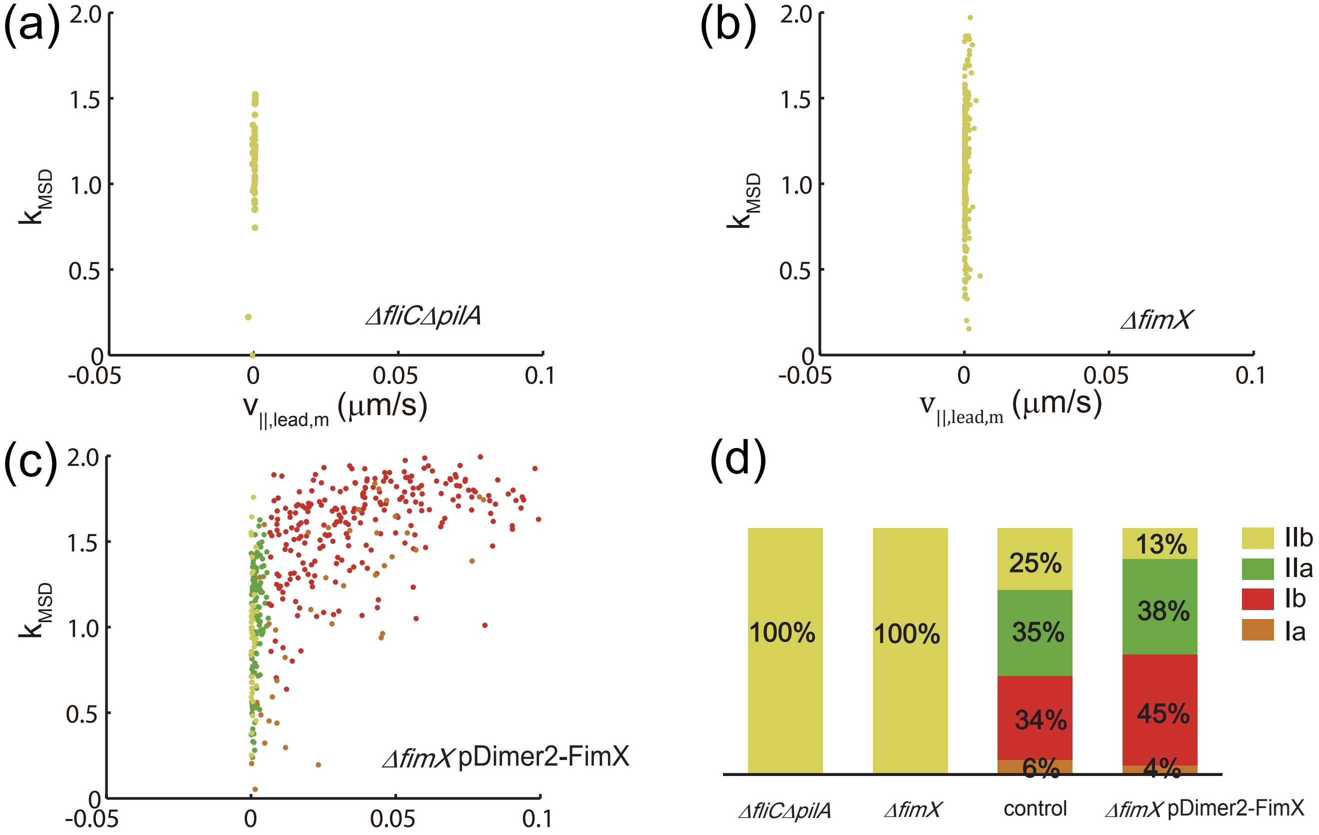

Supplementary Figure 4. Type Ia/b and IIa motilities require the expression of FimX. (*a* to *c*) Slope of the mean-squared displacement k_{MSD} as a function of median velocity along the cell body at the leading pole $v_{\|,\mathrm{lead,m}}$ for mutants (*a*) Δ*fli*CΔ*pil*A, (*b*) Δ*fim*X, and (*c*) Δ*fim*XpDimer2-FimX. (*d*) Subpopulations of twitching motility types in different mutants.

Supplementary Figure 5

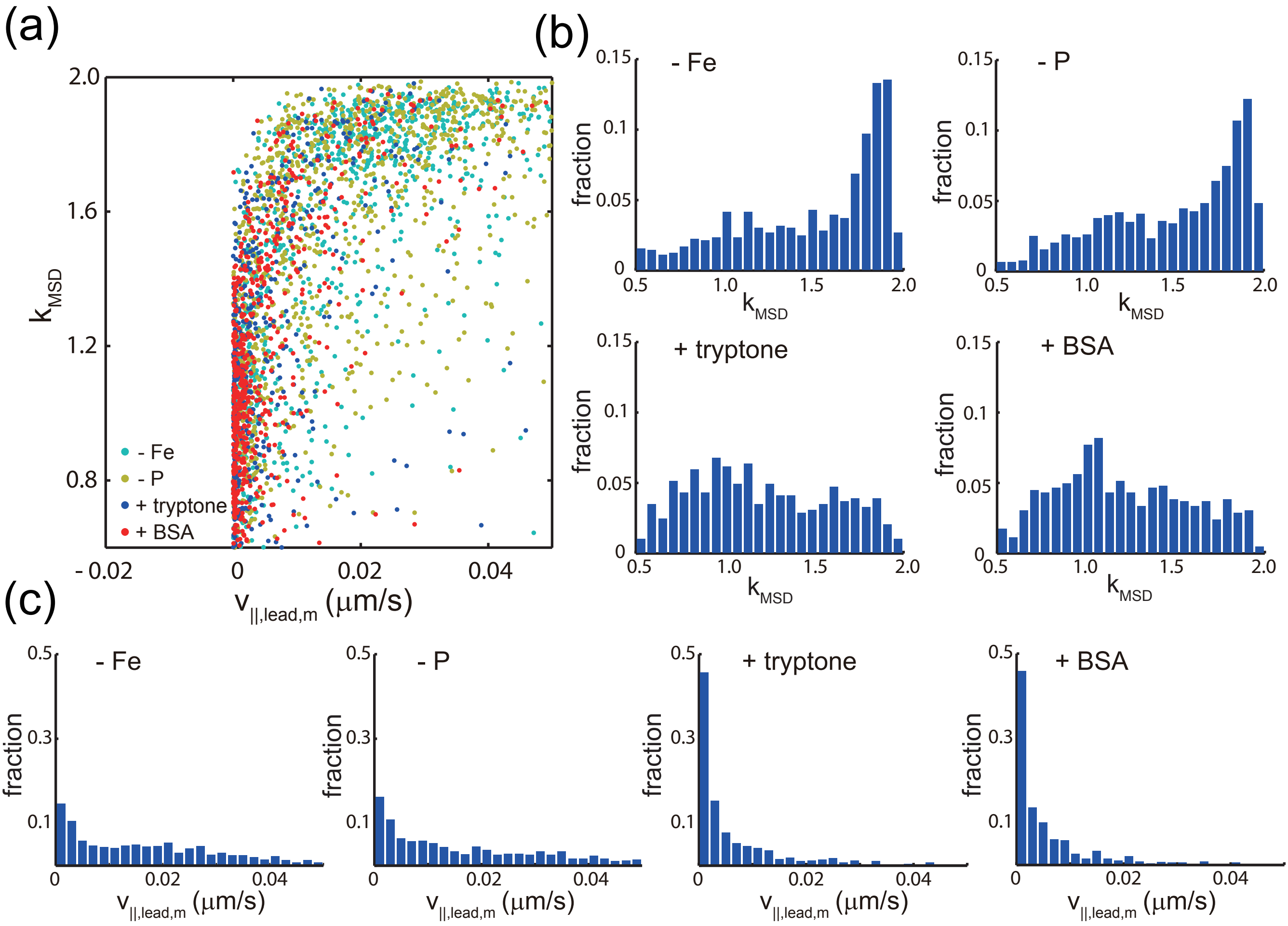

Supplementary Figure 5. Nutrient conditions affect the subpopulations of twitching motility types. (*a*) Slope of the mean-squared displacement k_{MSD} as a function of median velocity along the cell body at the leading pole $v_{\|,\mathrm{lead,m}}$. Cyan and olive symbols indicate nutrient-limited conditions (removing 90% phosphorus (-P) or removing 100% iron (-Fe)) and blue and red symbols indicate nutrient-supplemented condition (adding 0.1% (wt) BSA (+BSA) or adding 5% (wt) tryptone (+tryptone)). (*b*, *c*) Histograms of (*b*) k_{MSD} or (*c*) $v_{\|,\mathrm{lead,m}}$ in different nutrient conditions.

Supplementary Figure 6

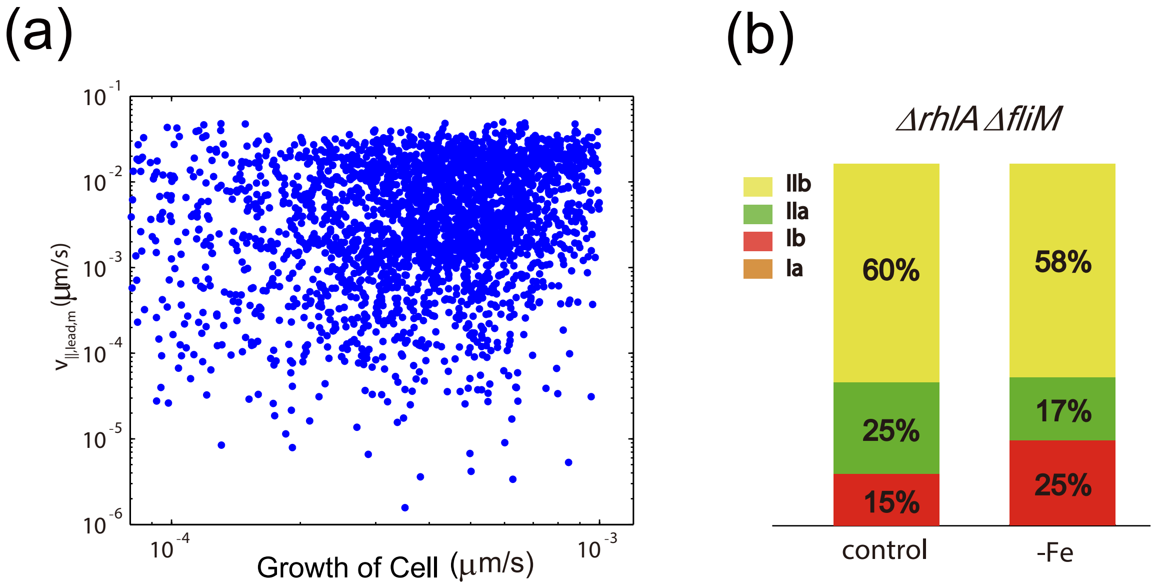

Supplementary Figure 6. (*a*) Median velocity along the cell body at the leading pole $v_{\parallel,\mathrm{lead,m}}$ as a function of the growth of cell at different nutrient conditions. (*b*) Subpopulations of twitching motility types in Δ*rhl*AΔ*fli*M mutant at different nutrient conditions.

Supplementary Figure 7

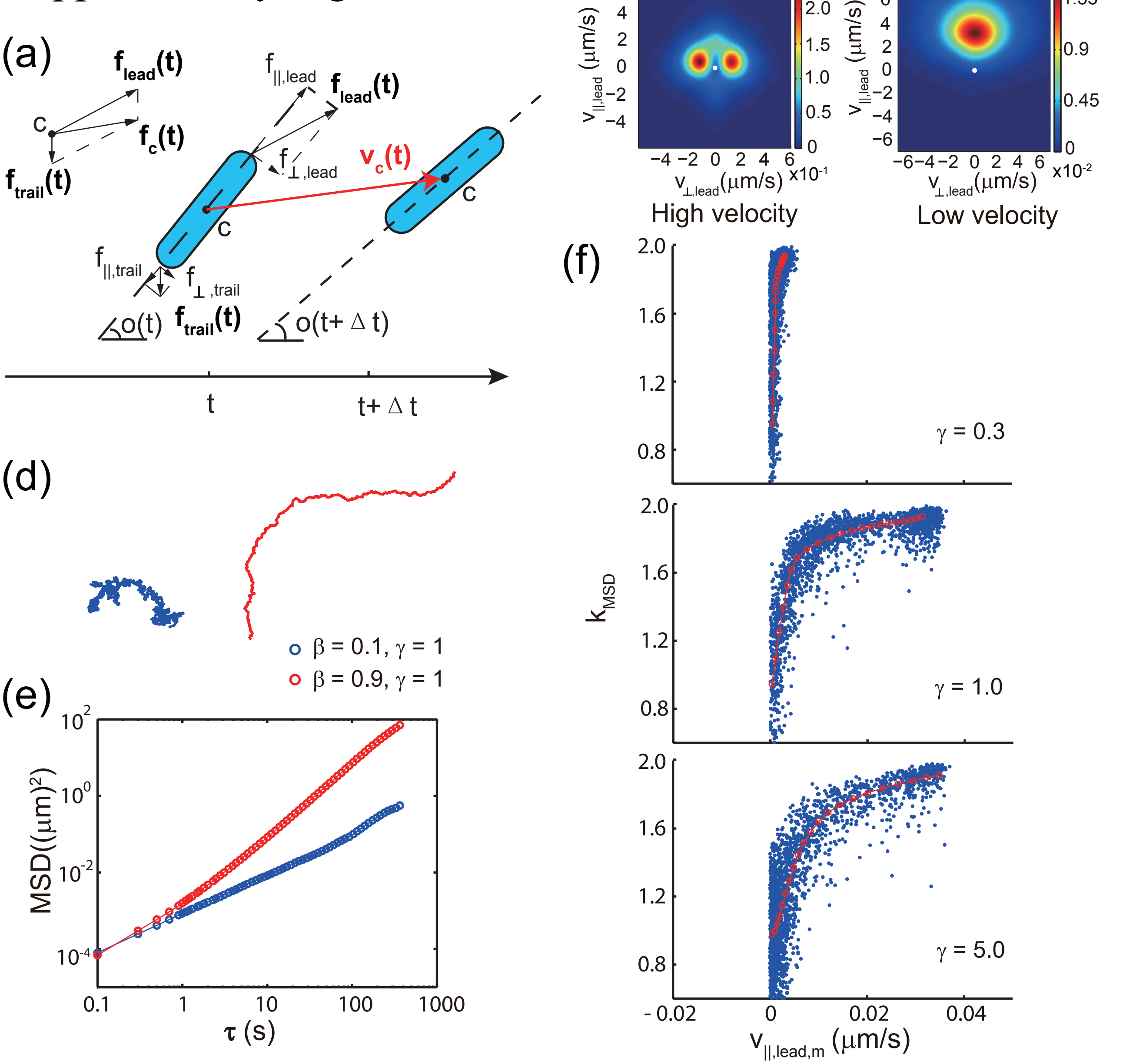

Supplementary Figure 7. Simulation of distinctive twitching motility types in single cells. (*a*) Schematic showing mechanical analysis of a bipolar-attached crawling cell. $\mathbf{f}_{\mathrm{lead}}(t)$ or $\mathbf{f}_{\mathrm{trail}}(t)$ indicates the instantaneous force that generated by pulling of TFP in the leading or the trailing pole, $f_{\parallel,\mathrm{lead}}, f_{\parallel,\mathrm{trial}}$ or $f_{\perp,\mathrm{lead}}, f_{\perp,\mathrm{trial}}$ is the component along or perpendicular to the body axis of the bacterium, $\mathbf{f}_{\boldsymbol{c}}(t)$ is the resultant force acting on the centroid, and $o(t)$ is the orientation of the cell body. In the simulation, a bacterium moves to a resultant position and orientation $o(t+\Delta t)$ at $t+\Delta t$ with a velocity ($\mathbf{v}_{\boldsymbol{c}}(t)$), where Δt is the time step. (*b*, *c*) Two-dimensional averaged distribution ($p(\mathbf{v}_{lead})$) of $\mathbf{v}_{lead}(t)$ in the 160 cells whose TFP only pull at their leading pole; (*b*) and (*c*) show the averaged distribution in the higher and lower ranges of velocity, respectively, and the colors indicate the magnitudes of the probability as indicated. (*d*) Simulated trajectories and (*e*) their MSD for parameters $\beta = 0.1, \gamma = 1$ (blue) or $\beta = 0.9, \gamma = 1$ (red). (*f*) Slope of the mean-squared displacement k_{MSD} as a function of median velocity along the cell body at the leading pole $v_{\parallel,\mathrm{lead,m}}$. Data points are obtained from 6,000 independent simulated trajectories in which (top) $\gamma = 0.3$, (middle), $\gamma = 1$, or (bottom) $\gamma = 5$ and β is varied from 0 to 1. Red lines indicate the ensemble-averaged results.

Supplementary Table 1

	Description	Source
**Strains**		
PAO1	wild type P. *aeruginosa* strain	J.D. Shrout
PAO1bΔ*fliM*	non-flagellated P. *aeruginosa* ATCC 15692 strain	J.D. Shrout
PAO1 Δ*fliC*Δ*pilA*	non-flagellated and non-piliate P. *aeruginosa* strain	J.J. Harrison
PAO1bΔ*rhlA*Δ*fliM*	*rhlA*::Gm^r derivative of non-flagellated *P. aeruginosa fliM*::Gm^r	(*1*)
PAO1 Δ*fimX*	*fimX*::Gm^r derivative of PAO1 (wild type)	This study
PAO1 pDimer2	PAO1 complemented with pDimer2	This study
PAO1Δ*fimX*pDimer2-FimX	PAO1Δ*fimX* complemented with pDimer2-FimX	This study
**Plasmid**		
pAK1900	Ap^r; multicopy E. coli-P. *aeruginosa* shuttle cloning vector	R. Sharp
pTdimer2-Golgi	Kan^r; functional *tdimer2* containing vector	GQ. Bi
pEX18Ap	Ap^r; gene replacement vector with MCS from pUC18	(*2*)
pPS858	Ap^r, Gmr; vector from pPS747	(*2*)
pEX18Ap-FimX	Ap^r; in-frame deletion of *fimX* constructed by PCR and cloned into EcoRI/HindIII sites of pEX18Ap	This study
pEX18Ap-FimXGm	Ap^r, Gmr; XbaI fragment from Pps858 containing the gentamicin-resistant cassette cloned into XbaI site of pEX18Ap-FimX	This study
pDimer2	Ap^r; *dimer2* fragment from pTdimer2-Golgi cloned into the HindIII-BamHI sites of PAK1900	This study
pFimX	Ap^r; a 2.8kb fragment containing *fimX* from PAO1 genome DNA cloned into the HindIII/BamHI sites of PAK1900	This study
pDimer2-FimX	Ap^r; *dimer2* fragment from pTdimer2-Golgi cloned into HindIII-NcoI sites of pFimX	This study
**Primers Sequence**		
FimXupEF	5'-CTAGAATTCATCCGCCAGGTACGCAATGGC-3'	
FimXupXR	5'-GATGGATCCTCTAGATTCCAGAATCAGCAGGCGG-3'	
FimXdnXF	5'-GATGGATCCTCTAGATTCTCCTCGGGAGACGAATG-3'	
FimXdnHR	5'-CGTGAAGCTTATCGCTTTCCAGCAGGACAG-3'	
Dimer2-HindIII-For	5'-GAGATAAGCTTATGGTGGCCTCCTCCGAGGACGT-3'	
Dimer2-BamHI-Rev	5'-GAGATGGATCCCTACAGGAACAGGTGGTGGCGG-3'	
FimX-HindIII-For	5'-GAGATAAGCTTGAGCAGAGCAGCCACTACCT-3'	
FimX-BamHI-Rev	5'-GAGATGGATCCCTGGGGATCGACTACGTGTT-3'	
Dimer2-BspHI-Rev	5'-GCTCATGAGGAACAGGTGGTGGCGG-3'	

Supplementary Table 1 Strains, plasmids, and primers used in this study.

Supplementary Methods:

Construction of the *P. aeruginosa* mutants. Three mutants, Δ*fimX*, pDimer2, and Δ*fimX*pDimer2-FimX, were constructed for this study. The upstream region of *fimX* (410bp) was amplified by PCR using primers FimXupEF (with EcoRI site) and FimXupXR (with XbaI site), while the downstream region (430bp) was amplified with primers FimXdnXF (with XbaI site) and FimXdnHR (with HindIII site) (Supplementary Tab. 1). After digestion by EcoRI/XbaI or HindIII/XbaI, respectively, the two DNA fragments were cloned into a gene replacement vector pEX18Ap (*2*) between EcoRI and HindIII restriction sites *via* a three-piece ligation. Next, the constructed plasmid pEX18Ap-fimX was digested with XbaI and then ligated with a gentamicin-resistant (Gm^r) cassette, which was obtained from Pps858 plasmid (*2*) by digesting with XbaI. The final constructed plasmid (pEX18Ap-FimXGm) was electroporated into PAO1 as described previously (*3*). The recombinant strain Δ*fimX* was identified by screening on LB agar plates containing 5% (w/v) sucrose with 30 μg/mL gentamicin, and further verified by PCR and sequencing. The *dimer2* fragment was amplified from pTdimer2-Golgi with primers Dimer2-HindIII-For and Dimer2-BamHI-Rev (Supplementary Tab. 1), and cloned into the HindIII-BamHI sites of plasmid PAK1900, allowing the *dimer2* to be expressed under the control of *lac* promoter. The plasmid pDimer2 was transformed into PAO1 to yield the strain pDimer2. A 2.8kb fragment containing *fimX* was amplified from PAO1 genome DNA using primers FimX-HindIII-For and FimX-BamHI-Rev (Supplementary Tab. 1), and cloned into the HindIII/BamHI digested PAK1900, which yielded plasmid pFimX. Then the *dimer2* fragment amplified from pTdimer2-Golgi using primers Dimer2-HindIII-For and Dimer2-BspHI-Rev was subcloned into the HindIII-NcoI sites of pFimX (Supplementary Tab. 1). The constructed plasmid pDimer2-FimX was transformed into Δ*fimX* to yield the strain Δ*fimX*pDimer2-FimX.

Expression and subcellular localization of FimX in P. ***aeruginosa*****.** Δ*fimX*pDimer2-FimX cells were first cultured for 24 hours on an LB agar plate at 37℃. Second, the cells were stored at 4 ℃ for 48 hours, allowing RFP to mature (4). Finally, cells were scraped from the agar plate and post-cultured at 27 ± 0.1℃ by flowing the corresponding media. A spinning-disk confocal microscope (Andor Revolution) equipped with a 100× oil objective was used to monitor the expression and subcellular localization of RFP-tagged FimX in single cells. RFP-tagged FimX was excited with a 561nm laser and the resulting fluorescence was collected through an emission filter (600 ± 25nm). Confocal images of dimension 512 pixels × 512 pixels were captured every ten seconds by an EMCCD (Andor iXon897) with 200 ms exposure time and 100×EM gain. Simultaneously, brightfield images (dimension 512 pixels × 512 pixels) of the cells were also collected. Switching of the acquisition channel between confocal and bright-field modalities was automatically controlled by commercial software (Andor IQ). Each dataset typically contained 3,600 brightfield/confocal images; cell contours were obtained from the brightfield images and the fluorescent intensity and subcellular localization of RFP-tagged FimX was obtained from the confocal images. Custom image processing and data analysis algorithms were developed in MATLAB.

Simulation of distinctive twitching motility types in single cells. The trajectory of centroid ($\mathbf{r}_c(t)$) and orientation ($o(t)$) of individual cells were obtained iteratively by $\mathbf{r}_c(t+\Delta t) = \mathbf{r}_c(t) + \mathbf{v}_c(t)\Delta t$ and $o(t+\Delta t) = o(t) + \omega(t)\Delta t$, starting with $\mathbf{r}_c = \mathbf{r_0}$ and $o = o_0$at $t = 0$. Here Δt is the time step, $\mathbf{v}_c(t)$ is the instantaneous velocity of the centroid, and $\omega(t)$ is the angular velocity of cell body. In the model, we make five assumptions:

(i) $\mathbf{v}_c(t)$ is linearly related to the net force ($\mathbf{f}_c(t) = \alpha\mathbf{v}_c(t)$ that is applied at the

centroid of cell body, where α is the averaged drag coefficient.

(ii) $\mathbf{f}_c(t)$ is the resultant force driven by pulling of TFP at the leading or trailing pole ($\mathbf{f}_{lead}(t) + \mathbf{f}_{trail}(t)$).

(iii) The probability that TFP pull at the leading or trailing poles ($p_{\text{lead,g}}$ or $p_{\text{trail,g}}$) is positively correlated to the local concentration of FimX at the leading or trailing pole (c_{lead} , c_{trail}), namely, $p_{\text{lead,g}} = 1 - \exp(-\, c_{\text{lead}}/c^*)$ and $p_{\text{trail,g}} = 1 - \exp(-\, c_{\text{trail}}/c^*)$, where c^* is the characteristic concentration of FimX that enables the assembly of TFP.

(iv) The average subcellular concentration of FimX (c_t) is independent of time. Note that $2c_t = c_{\text{lead}} + c_{\text{trail}}$ because subcellular localization of FimX is either unipolar or bipolar, and thus the symmetry parameter $\beta \equiv (c_{\text{lead}} - c_{\text{trail}})/2c_t$ describes the subcellular localization of FimX. Similarly, $\gamma \equiv c_t/c^*$ describes the expression of FimX in single cells. Using these definitions, $p_{\text{lead,g}}$ and $p_{\text{trail,g}}$ can be rewritten as functions of β and γ : $p_{\text{lead,g}} = 1 - \exp[-\gamma(1+\beta)]$ and $p_{\text{trail,g}} = 1 - \exp[-\gamma(1-\beta)]$.

(v) $\omega(t)$ is determined by $(f_{\perp,\text{lead}} - f_{\perp,\text{trial}})/\alpha$, where $f_{\perp,\text{lead}}$ or $f_{\perp,\text{trial}}$ is the force component of $\mathbf{f}_{\text{lead}}(t)$ or $\mathbf{f}_{\text{trail}}(t)$ normal to the major axis of cell body. The model is schematically shown in Supplementary Fig. 7*a*

Next, the force distributions ($p(\mathbf{f}_{\text{lead}})$ or $p(\mathbf{f}_{\text{trail}})$) were evaluated from the distribution of instantaneous velocity $p(\mathbf{v}_{\text{lead}})$ because $\mathbf{f}_{\text{lead}}(t) = \alpha\mathbf{v}_{\text{lead}}(t)$ in unipolar attached crawling cells. Therefore, either $\mathbf{f}_{\text{lead}}(t)$ or $\mathbf{f}_{\text{trail}}(t)$ could be sampled from $\alpha p(\mathbf{v}_{\text{lead}})$ given a certain $p_{\text{lead,g}}$ or $p_{\text{trail,g}}$ using Monte Carlo method, and $p(\mathbf{v}_{\text{lead}})$ could be directly obtained from experimental data, as shown in Supplementary Fig. 7*b* and *c*. Because *P. aeruginosa* cells can use TFP to slingshot

on surfaces (5), slower and faster velocities were separately sampled, and a threshold $v_{\|,\mathrm{lead,m}} > 5\sigma_{\|,\mathrm{lead}}$ was used to identify the slingshot motion of each single cell. Finally, simulated trajectories for each pole ($\mathbf{r}_{\mathrm{lead}}(t)$ or $\mathbf{r}_{\mathrm{trail}}(t)$) were calculated from $\mathbf{r}_c(t)$ and $o(t)$. The resulting $\mathbf{r}_{\mathrm{lead}}(t)$, $\mathbf{r}_{\mathrm{trail}}(t)$ and $\mathbf{r}_c$ depended only on β and γ. Supplementary Fig. 7d and e show simulated trajectories generated using ($\beta = 0.1, \gamma = 1$) and ($\beta = 0.9, \gamma = 1$), respectively. Simulated trajectories $\mathbf{r}_{lead}(t)$ and $\mathbf{r}_{trail}(t)$ were analyzed using the same methods and criteria established for the four motility types in the experiments. Finally, the bivariate dependence (β and γ) of the ensemble average of k_{MSD} or $v_{\|,\mathrm{lead,m}}$ ($< k_{\mathrm{MSD}} >$ or $< v_{\|,\mathrm{lead,m}} >$) was directly calculated by averaging of 6000 independent simulation results.

Searching efficiency of single cells. Target sites in the model were randomly distributed ($\mathbf{r}_T$) on the surface with a certain density (ρ_s). In the simulation, single cells moved on the surface according to their simulated trajectories $\mathbf{r}_c(t|\beta,\gamma)$ as described above. The first successful searching time of any target site t_s was defined as $|\mathbf{r}_T - \mathbf{r}_c(t = t_s|\beta,\gamma)| < l_B$, where l_B is the length of cell. The searching efficiency (η), which was a function of both β and γ, was defined as

$$\eta_s(\beta,\gamma) = \frac{\Delta t}{<t_s>} \qquad (1)$$

where $< t_s >$ is the ensemble average of t_s. η_s was further normalized by multiplying a factor $\lambda = 1/(l_B^2\rho_s)$, so that $\eta_s\lambda$ could be used to compare the searching efficiencies at various ρ_s (6).

Clustering efficiency of single cells. In the simulation, single cells moved on the surface according to their simulated trajectories $\mathbf{r}_c(t|\beta,\gamma)$ as described above. In addition, the division of cell was allowed in a certain doubling time (t_d). After division, the trajectories of daughter cells

were resampled from $\mathbf{r}_c(t|\beta = \beta_m, \gamma = \gamma_m)$, where β_m and γ_m are the parameters of their mother. After a fixed time period ($t = 7\ t_d$), cells separated by less than a length threshold l_B were determined to be in a cluster. We therefore defined the clustering efficiency (η_c), which was a function only of β and γ, as

$$\eta_c(\beta, \gamma) = < \frac{N_c}{N_{all}} > \quad (2)$$

where N_c is the average number of cells in clusters and N_{all} is the total number of cells.

Supplementary References

1. Zhang R. R. , Ni L. , Jin Z. Y. , Li J. H. & Jin F. Bacteria slingshot more on soft surfaces. *Nat. Commun.* **5**, 6 (2014).
2. Hoang T. T. , Karkhoff-Schweizer R. R. ,Kutchma A. J. , Schweizer H. P. , A broad-host-range Flp-FRT recombination system for site-specific excision of chromosomally-located DNA sequences: application for isolation of unmarked Pseudomonas aeruginosa mutants. *Gene* **212**, 77-86 (1998).
3. Choi K. H. , Kumar A. , Schweizer H. P. , A 10-min method for preparation of highly electrocompetent Pseudomonas aeruginosa cells: Application for DNA fragment transfer between chromosomes and plasmid transformation. *J. Microbiol. Methods* **64**, 391-397 (2006).
4. Huang B. X. , Whitchurch C. B. , Mattick J. S. , FimX, a multidomain protein connecting environmental signals to twitching motility in Pseudomonas aeruginosa. *J. Bacterial.* **185**, 7068-7076 (2003).
5. Jin F. ,Conrad J. C. , Gibiansky M. L. , Wong G. C. L. , Bacteria use type-IV pili to slingshot on surfaces. *Proc. Natl. Acad. Sci. USA.* **108**, 12617-12622 (2011).
6. Viswanathan G. M. *et al.*, Optimizing the success of random searches. *Nature* **401**, 911-914 (1999).
