# Supplementary material for: Bacteria differently deploy type-IV pili on surfaces to adapt to nutrient availability
Source: NPJ Biofilms Microbiomes. 2016 Feb 24;2:15029–. doi: 10.1038/npjbiofilms.2015.29 (PMC5515259; doi:10.1038/npjbiofilms.2015.29)
Supplement: Supplementary Movie 1 and 2 Legends [file npjbiofilms201529-s12.pdf]

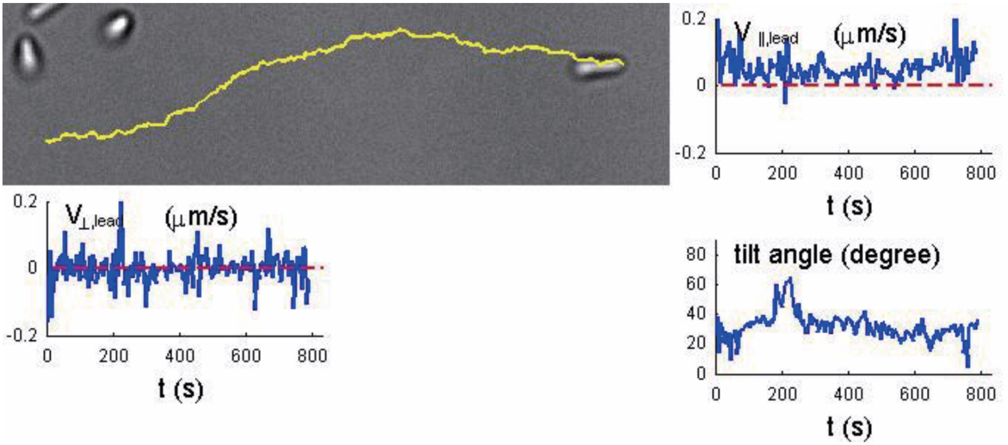

Movie S1. Unipolar-attached crawling cell (Type Ia) on glass surface. Subpanels show the time series of $v_{\parallel,\mathrm{lead}}(t)$, $v_{\perp,\mathrm{lead}}(t)$ and tilt angle.

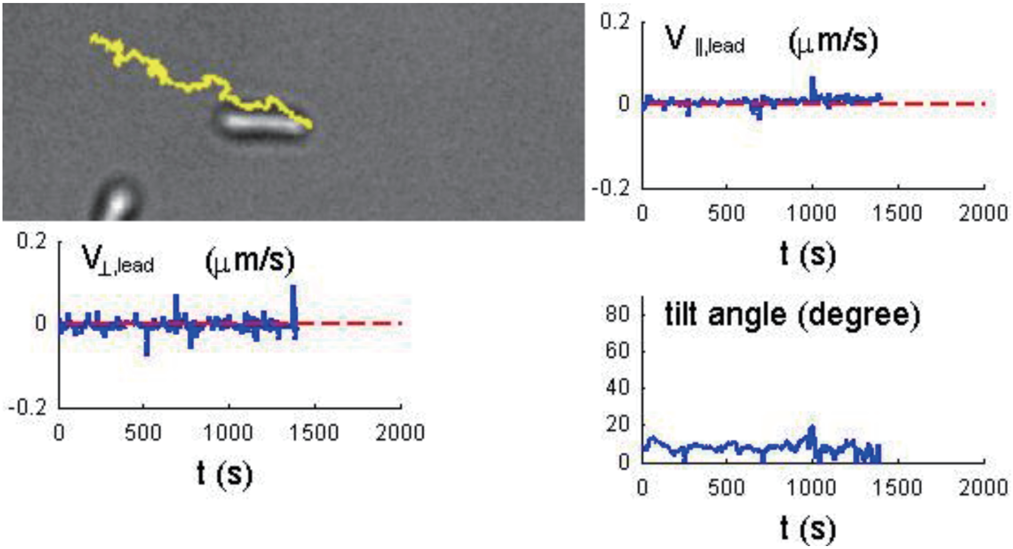

Movie S2. Bipolar-attached crawling cell (Type Ib) on glass surface. Subpanels show the time series of $v_{\parallel,\mathrm{lead}}(t)$, $v_{\perp,\mathrm{lead}}(t)$ and tilt angle.
