# Supplementary material for: Bacteria differently deploy type-IV pili on surfaces to adapt to nutrient availability
Source: NPJ Biofilms Microbiomes. 2016 Feb 24;2:15029–. doi: 10.1038/npjbiofilms.2015.29 (PMC5515259; doi:10.1038/npjbiofilms.2015.29)
Supplement: Supplementary Movie 3 and 4 Legends [file npjbiofilms201529-s13.pdf]

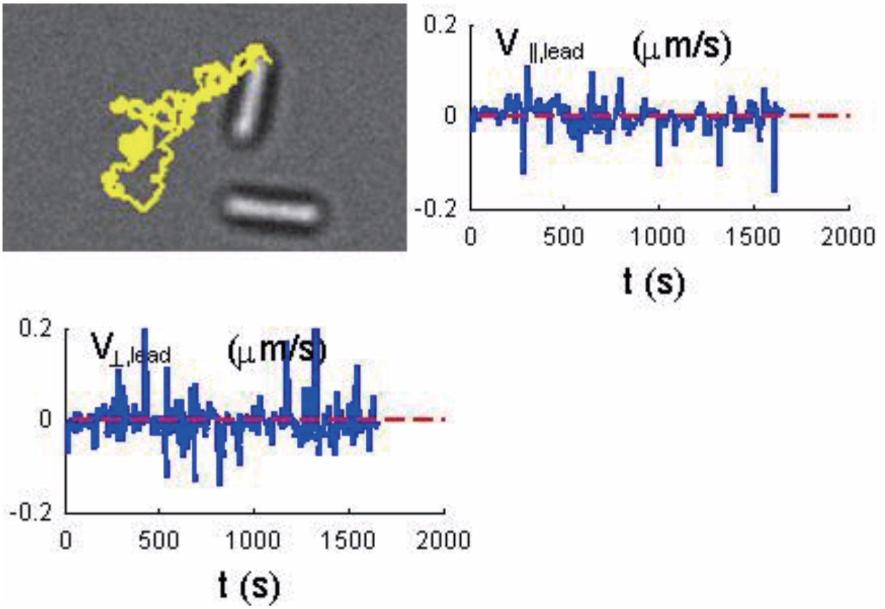

Movie S3. Bipolar-attached wiggling cell (Type IIa) on glass surface. Subpanels show the time series of $v_{\parallel,\text{lead}}(t)$, $v_{\perp,\text{lead}}(t)$.

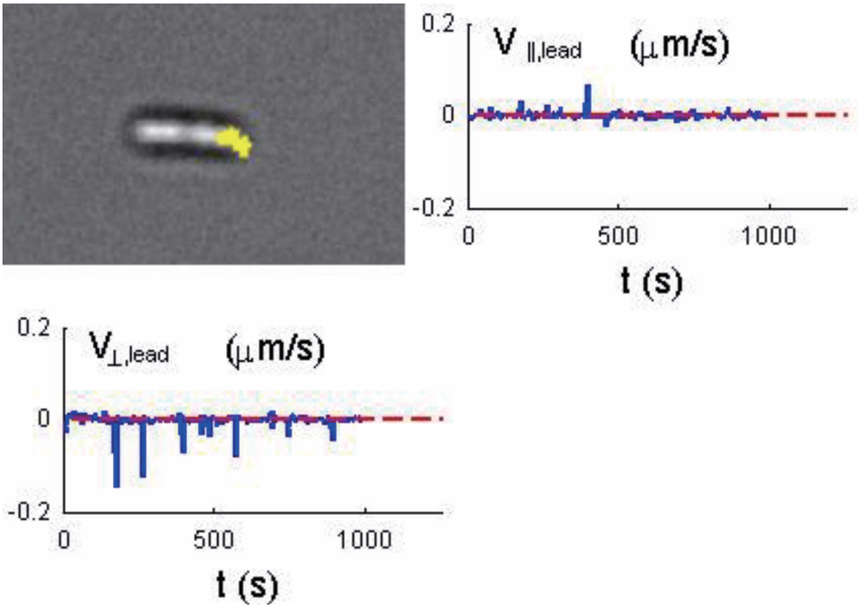

Movie S4. Bipolar-attached stalling cell (Type IIb) on glass surface. Subpanels show the time series of $v_{\parallel,\text{lead}}(t)$, $v_{\perp,\text{lead}}(t)$.
