# Supplementary material for: Bacteria differently deploy type-IV pili on surfaces to adapt to nutrient availability
Source: NPJ Biofilms Microbiomes. 2016 Feb 24;2:15029–. doi: 10.1038/npjbiofilms.2015.29 (PMC5515259; doi:10.1038/npjbiofilms.2015.29)
Supplement: Supplementary Movie 5 and 6 Legends [file npjbiofilms201529-s14.pdf]

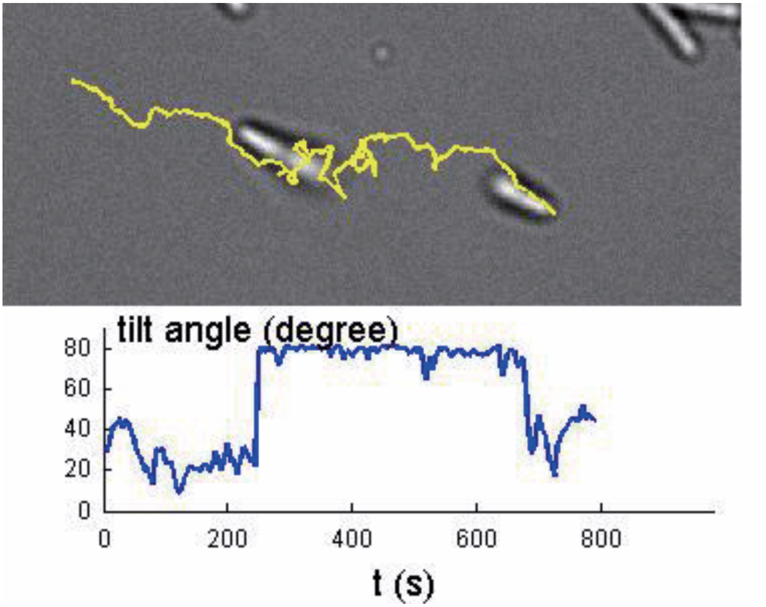

Movie S5. Unipolar-attached cell switches its motility between the crawling and the walking. Subpanels show the time series of tilt angle.

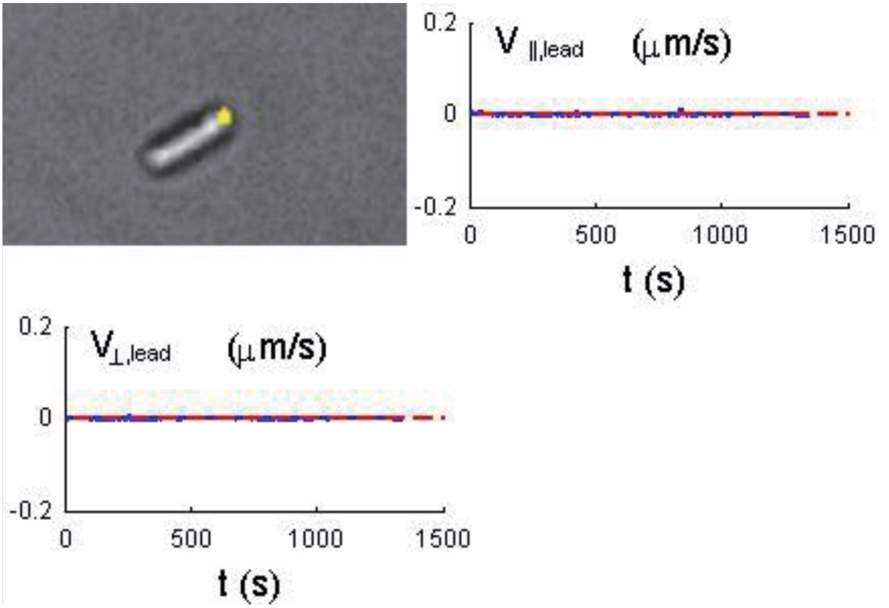

Movie S6. Δ*fli*CΔ*pil*A cell (non-motile phenotype) on glass surface. Subpanels show the time series of $v_{\parallel,\text{lead}}(t)$, $v_{\perp,\text{lead}}(t)$.
