# Supplementary material for: Bacteria differently deploy type-IV pili on surfaces to adapt to nutrient availability
Source: NPJ Biofilms Microbiomes. 2016 Feb 24;2:15029–. doi: 10.1038/npjbiofilms.2015.29 (PMC5515259; doi:10.1038/npjbiofilms.2015.29)
Supplement: Supplementary Movie 7 and 8 Legends [file npjbiofilms201529-s15.pdf]

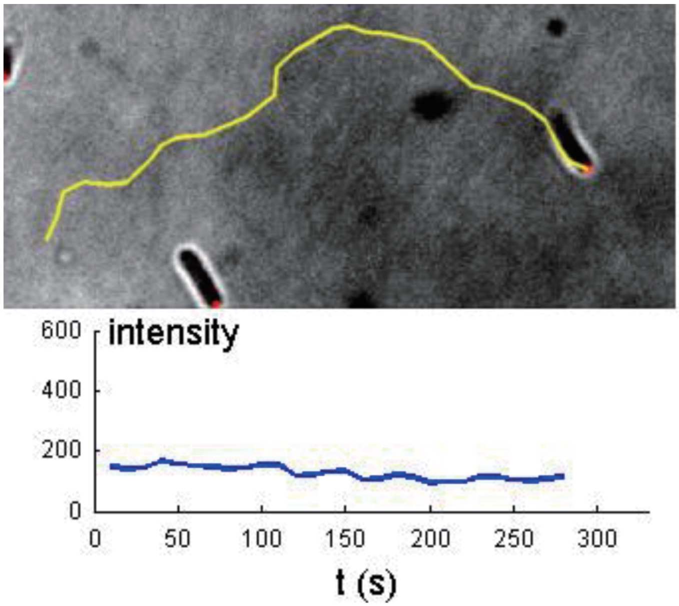

Movie S7. RFP-tagged FimX in unipolar-attached crawling cell (Type Ia) on glass surface. Subpanel shows the time series of fluorescent intensities of FimX.

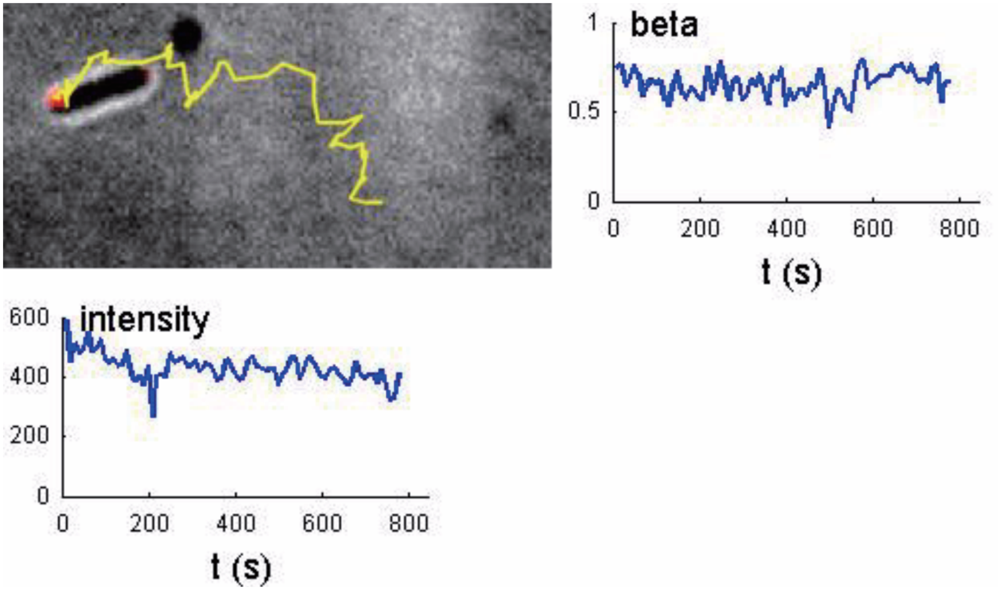

Movie S8. RFP-tagged FimX in bipolar-attached crawling cell (Type Ib) on glass surface. Subpanels show the time series of fluorescent intensities and symmetry parameter (β) of FimX.
