# Supplementary material for: Bacteria differently deploy type-IV pili on surfaces to adapt to nutrient availability
Source: NPJ Biofilms Microbiomes. 2016 Feb 24;2:15029–. doi: 10.1038/npjbiofilms.2015.29 (PMC5515259; doi:10.1038/npjbiofilms.2015.29)
Supplement: Supplementary Movie 9 and 10 Legends [file npjbiofilms201529-s16.pdf]

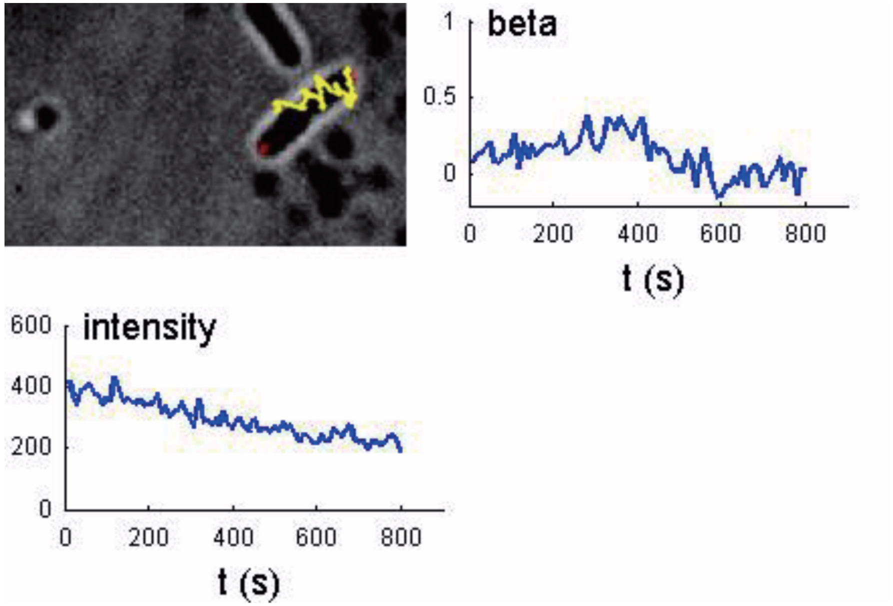

Movie S9. RFP-tagged FimX in bipolar-attached wiggling cell (Type IIa) on glass surface. Subpanels show the time series of fluorescent intensities and symmetry parameter (β) of FimX.

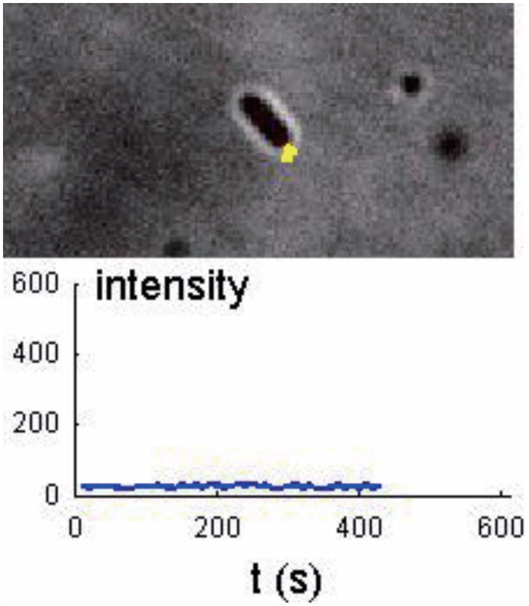

Movie S10. RFP-tagged FimX in bipolar-attached stalling cell (Type IIb) on glass surface. Subpanel shows the time series of fluorescent intensities.
